# Uncovering the fragmentation and separation characteristics of sophorolipid biosurfactants with LC-MS-ESI

**DOI:** 10.1093/jimb/kuae035

**Published:** 2024-09-26

**Authors:** Benjamin Ingham, Katherine Hollywood, Phavit Wongsirichot, Alistair Veitch, James Winterburn

**Affiliations:** Department of Chemical Engineering, The University of Manchester, Oxford Road, Manchester M13 9PL, UK; Manchester Institute of Biotechnology, Department of Chemistry, The University of Manchester, Manchester M1 7DN, UK; Department of Chemical Engineering, The University of Manchester, Oxford Road, Manchester M13 9PL, UK; Holiferm Ltd., Unit 15, Severnside Trading Estate, Textilose Road, Manchester M17 1WA, UK; Department of Chemical Engineering, The University of Manchester, Oxford Road, Manchester M13 9PL, UK

**Keywords:** Sophorolipid, Mass spectrometry, Fragmentation, Glycolipids

## Abstract

The application of liquid chromatography and mass spectrometry (MS) is a challenging area of research for structural identification of sophorolipids, owing to the large number of possible variations in structure and limited knowledge on the separation and fragmentation characteristics of the variants. The aims of this work was to provide a comprehensive analysis of the expected characteristics and fragmentation patterns of a wide range of sophorolipid biosurfactant congeners, providing a methodology and process alongside freely available data to inform and enable future research of commercial or novel sophorolipids. Samples of acidic and lactonic sophorolipid standards were tested using reverse-phase ultra-high performance liquid chromatography and identified using electrospray ionization MS. 37 sophorolipid variants were identified and compared for their elution order and fragmentation pattern under MS/MS. The retention time of sophorolipids was increased by the presence of lactonization, unsaturation, chain length, and acetylation as hydrophobic interactions with the C18 stationary phase increased. A key finding that acidic forms can elute later than lactonic variants was obtained when the fatty acid length and unsaturation and acetylation are altered, in contradiction to previous literature statements. Fragmentation pathways were determined for lactonic and acidic variants under negative [M–H]^−^ and positive [M+NH_4_]^+^ ionization, and unique patterns/pathways were identified to help determine the structural components present. The first publicly available database of chromatograms and MS2 spectra has been made available to aid in the identification of sophorolipid components and provide a reliable dataset to accelerate future research into novel sophorolipids and shorten the time to innovation.

**One-Sentence Summary:**

This article describes the process and challenges in identifying different structures of eco-friendly biosurfactants, providing a novel database to compare results.

## Introduction

Sophorolipids (SLs) are a form of glycolipid biosurfactant produced by the yeast *Starmerella bombicola* that possess high surface activity alongside low toxicity and biodegradability, making them an attractive alternative to petrochemically derived surfactants (Develter & Lauryssen, 2010; Hirata et al*.*, 2009; Li et al*.*, 2020; Ma et al., 2012). Advances in genetic engineering, bioreactor design, feedstock utilization, and development of alternative feedstocks are enabling higher yields from of SL production, lowering manufacturing cost, reducing environmental impact, and allowing competition with low-cost petrochemical surfactants (Dolman et al*.*, 2017; Jiménez-Peñalver et al*.*, 2019; Lin et al*.*, 2019; Liu et al*.*, 2022; Wongsirichot et al., 2021). For every process improvement that is attempted, the final composition and quantity of SLs (and specific available congeners) must be found using an appropriate analytical method. This presents a challenge, as fermentation samples typically host a mixture of SLs with a range of structural variances, meaning characterization requires broad techniques that can handle the different physiochemical properties of these variants to separate and positively identify the structure. Developing these broad techniques is difficult, and well characterized and reproducible methods are not abundantly available, leading to a bottleneck in process innovation and the commercialization of SL biosurfactants.

**Fig. 1.**
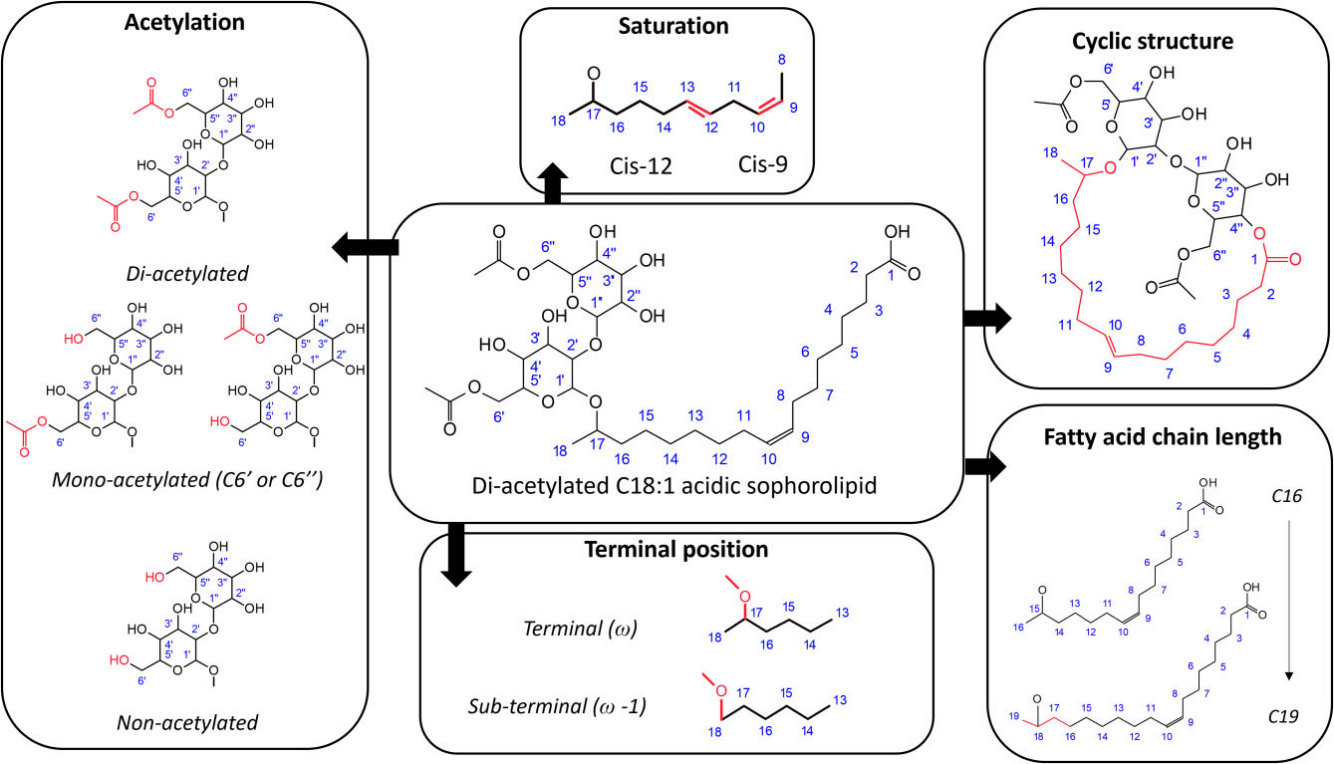
Overview of the structural changes that can occur in a sophorolipid. With C18:1 diacetylated acidic sophorolipid as the base molecule, changes occur in the acetylation, terminal hydroxylation position, fatty acid chain length, cyclic structure (to “lactonic”), and fatty acid unsaturation (mono- or diunsaturated).

Figure 1 describes the composition of SLs and structural variations that can occur during the production process. Sophorolipids are composed of a dimer of glucoses bound by a β1′-2″ bond (′ = first bound glucose attached to the fatty acid, ″ = the secondary glucose attached to the first bound glucose unit) to form a sophorose unit (2-O-β-d-glucopyranosyl-d-glucopyranose), which is linked to a hydrophobic terminal/subterminal hydroxylated fatty acid group via a glycosidic bond at C1′. Structurally, the SL can appear in a variety of forms (Fig. 1). The major structural variations are seen in the carboxylic acid of the fatty acid chain, appearing as acidic (free carboxylic acid) or lactonic (internal esterification between the carboxyl group and C4″ of the sophorose) forms (Saerens et al*.*, 2011). On the sophorose acetylation occurs primarily on the 6′ or 6″ position (for non- [NA], mono- [MA], or diacetylated [DA] forms) (Asmer et al*.*, 1988). In the hydroxylated fatty acid chain, variations occur in the fatty acid length (typically 16–18 carbons), unsaturation (at *cis-*9 and *cis-*12), and position of the hydroxyl group (terminal or subterminal) (Van Bogaert et al*.*, 2007b).

The large number of sites that can be altered in the chemical structure of SLs leads to numerous potential structures that can be produced by the wild-type yeast strain alone. Between C16- and C19-length fatty acid chain, there are 144 potential variants, with some more common than others due to the preferences of production-related enzymes (Huang et al., 2014; Saerens et al*.*, 2011). With increasing research in genetic editing and novel feed applications, the number of potential SL structures is becoming even larger, with SLs being produced with short- or long-chain fatty acids and novel “bolaforms” (Abhyankar et al*.*, 2021; Dierickx et al*.*, 2022; Kumari et al*.*, 2021; Van Bogaert et al*.*, 2016; Zerhusen et al*.*, 2020).

Whilst there are methods, such as liquid–liquid extraction, that can roughly quantify the total amount of SL present in a sample (e.g., produced from a fermentation process), having an understanding of the specific structural composition is important in both research and industrial processes as differences can lead to strikingly different physiochemical characteristics and applications. Different variants have different characteristics that are favoured in different applications. For example, alteration of the ring structure causes a change in the surface activity and foamability of SLs, whilst reducing the length of the fatty acid chain can improve emulsification activity (Ahn et al., 2016; Hirata et al*.*, 2009). The ability to identify desired SL forms allows for targeted process optimization and can subsequently improve the cost-effectiveness of the production process. Moreover, industrial production processes must demonstrate the specific composition of all material to maintain regulatory compliance and enter/remain in markets, placing an emphasis on having an accurate and specific method for identifying SL structures.

Amongst the most popular tool for SL analysis is liquid chromatography (LC), which uses the hydrophobicity and polarity of analytes to separate and identify SL variants. The close physiochemical characteristics of SL variants makes separation challenging, with a need to use gradient elution conditions with a reverse-phase (RP) C18 column in order to separate closely related variants (Davila et al*.*, 1993). From this separation, detectors applying evaporative light scattering detection (ELSD) present a variety of peaks that correspond to the components in the fermentation broth, including the SLs. Liquid chromatography does not provide information on the structural form of the peaks present in the chromatogram and requires prior knowledge of the expected relative position of each structural variant to confidently identify the composition of the sample. Unfortunately, standards of SL variants are not widely available and those that are available are not pure/variant specific. Some knowledge of the general separation of SLs on RP C18 columns has been suggested, for example with acidic forms eluting first, but the specific elucidation order has not been investigated and there has been no research into where structural variants may cross over or how the other structural variants (chain length, unsaturation, and acetylation) affect the elution order of peaks. To identify structural variants, mass spectrometry (MS) can be used to ionize the eluted structures and provide information on the molecular mass and intensity. Electrospray ionization (ESI) is most commonly used for SL identification, as the “soft” ionization process generates ions with low fragmentation, allowing for accurate determination of the molecular mass. From this, tandem MS (MS2) can be applied to selected ions for controlled fragmentation, providing structural information of the compound.

The accuracy of the MS method applied is extremely important when attempting to identify SL variants, particularly as different forms can share similar masses. For example, the monoisotopic mass of lactonic DA C16:1 (660.33571 Da) and lactonic MA C19:1 (660.372095 Da) only differs by 0.04 Da, so accurate detection and reporting is incredibly important. Despite this, a significant portion of the literature only reports to a whole unit or one decimal place in their mass identification, weakening confidence in the identification. Even with accurate measurement, some variants have identical mass, particularly when comparing acidic and lactonic compounds, with lactonic DA C17:0 and acidic MA C19:0 (C_33_H_56_O_14_) both showing a neutral mass of 675.359734 Da. Similarly, variants attached to different adducts can produce identical masses; the adducts of [M+CHO_2_]^−^ and [M+CH_3_CO_2_]^−^ both differ by a mass of 14.016747 Da (a change of CH_2_), which is identical to the mass change found when the fatty acid chain of the structure increases by one carbon. This could potentially lead to selection of adducts [lactonic DA C19:1+CHO_2_]^−^ and [lactonic DA C18:1+CH_3_CO_2_]^−^, which both have a mass of 747.38086 Da and to the assumption that the selected forms are identical structures. These example cases focus solely on SLs, which are typically only part of the analytes found in a sample derived from a complex fermentation media that is poorly defined (i.e., using yeast extract, corn steep liquor, etc.) that can contain components that may share the same mass as some of the SL variants. Confident identification of SL structure with LC-MS is a challenge that encompasses consideration of the separation, ionization, and fragmentation characteristics of different variants and requires understanding of how different structural changes alter these characteristics.

In this work, LC-MS-ESI and tandem MS with higher-energy collisional dissociation (HCD) have been used to separate and identify SL variants with a wide range of structures. The paper presents a walkthrough and considerations of initial peak selection using the various adduct forms. Fragmentation with tandem MS is then explored, supported by in silico fragmentation predictions, providing fragmentation pathways and a guide to key fragments that relate to alterations of the SL structure, with a publicly available database of 71 MS2 spectra available for further research. These positively identified SL structures were then linked back to the retention time of the variants and the expected elucidation order, with crossover of major structural components explored to better understand the separation behaviour of SLs in a standard RP gradient high-performance liquid chromatography (HPLC) method; a publicly available database of chromatogram separations is available. This work expands knowledge of SL separation and identification with LC-MS and provides databases to accelerate application of these techniques to SL development, identifying key fragmentation pathways and patterns to confidently identify the specific SL congener found and aid in the process of identification of novel compounds.

## Materials and Methods

### Materials

Standards of C18:1 DA lactonic SL, C18:1 “mix” acetylated acidic SL, and C18:1 NA acidic SL were acquired from Biosynth (U.K.). A further C18:1 DA lactonic SL standard was acquired from Biobase Europe (Belgium). Sulfuric acid, pure ethanol, LC-MS grade water, and acetonitrile were acquired from Fisher Scientific (U.K.).

### Naming Conventions

To improve readability, the following naming convention is used to identify the specific SL variant based on their structure, as shown in Table 1. The naming structure is composed as follows:


\begin{eqnarray*}
\left[ {{\mathrm{Carbon\ number}}} \right]:\left[ {{\mathrm{saturation}}} \right] + \left[ {{\mathrm{acetylation}}} \right]{\mathrm{\ }} +\nonumber\\
\left[ {{\mathrm{hydroxylation\ position}}} \right]{\mathrm{\ }} + {\mathrm{\ }}\left[ {{\mathrm{chain\ structure}}} \right]
\end{eqnarray*}

**Table 1.** Naming Convention for Sophorolipid Structural Components

**Saturation**	
Saturated	0
Monounsaturated	1
Diunsaturated	2
**Acetylation**	
Nonacetylated	NA
Monoacetylated C6′	MA C6′
Monoacetylated C6″	MA C6″
Diacetylated	DA
**Hydroxylation position**	
Subterminal	ω-1
Terminal	Ω
**Chain structure**	
Lactonic (closed)	Lac
Acidic (open)	Acid

For example, 18-carbon monounsaturated DA subterminal hydroxylated lactonic is notated “C18:1 DA ω-1 Lac.” The hydroxylation position is omitted in cases where it is unknown.

### LC-MS Identification of Sophorolipid Variant

#### Identifying targets with a predictive database

To correctly identify and calculate the molecular formula and monoisotopic mass of possible combinations of SL structures, the Custom Design tool from JMP 17 was used to compile a database containing combinations of each structural characteristic, combining the fatty acid chain length (16, 17, 18, or 19), unsaturation (0, 1, or 2), acetylation (non, mono-, or di-), and chain form (acidic or lactonic) to create 72 possible combinations. Structural variants were drawn in ChemSketch (ACD Labs, Canada) and their molecular formula calculated. These formulae were then used to create a generalized linear model that would calculate the number of C, H, and O atoms of any variants within the range of the structural characteristics. From this, the monoisotopic mass of C, H, and O was used to determine an accurate monoisotopic mass for each variants to six decimal points. Accurate masses of common negative and positive adduct forms were also calculated (for access to the database, see Supplementary Information).

#### Structure and characteristics of sophorolipid variants

Structures were generated using ChemSketch (ACD Labs, Canada) for all SL variants in order to generate InChI keys for use in the in silico fragmentation tool MetFrag and to provide structures for databasing. The structures were based on a set range of structural variants in the fatty acid length (C16–C19), number of unsaturations (0, 1, or 2), number of acetyl groups (NA, MA, or DA), and an open (acidic) or closed ring (lactonic) form. Structural variation positions were based on the most common forms seen in the literature, with unsaturation occurring on *cis-*9 (monounsaturated) and/or *cis-*12 (diunsaturated), glycosidic bonds on C2′ and C1″, carboxylic acid esterification at C4″, and acetylation on C6′ and C6″ (Asmer et al*.*, 1988; Saerens et al*.*, 2011; Van Bogaert et al*.*, 2007a). LogP values for the variants were also calculated using ChemSketch.

#### LC-MS conditions

To confirm the structure and retention time of SL variants, analysis was performed on a Q Exactive Plus equipped with an ESI probe coupled to an Ultimate 3000 UHPLC. The UHPLC was equipped with an Accucore Vanquish C18+ RP column (2.1 mm × 100 mm × 1.5 µm). LC-MS grade water and acetonitrile (ACN) were used as mobile phases, with 0.1% formic acid added to both. Flow rate was 0.3 mL/min with an exploratory gradient (low to high) of 2% ACN for 3 min, 2–98% ACN for 42 min, 98% ACN for 10 min, 98–2% ACN for 0.1 min, and equilibration at 2% ACN for 4.9 min for a total run time of 60 min. The column was kept at 50 °C and samples were chilled in the autosampler to 10 °C; 5 µL of sample was injected onto the column.

Data acquisition was conducted in full scan mode with data-dependent acquisition for the top five most abundant ions per scan. The scan range was 100–1,500 m/z with a resolution of 70,000 and an automatic gain control (AGC) target of 3 × 10^6^ and a maximum integration time of 200 ms. Data-dependent MS2 acquisition was undertaken with a resolution of 35,000 and an AGC target of 5 × 10^4^, a maximum integration time of 50 ms, a loop count of 5, an isolation window of 1 m/z, and a collision energy of 20 eV. The acquisition was conducted in both positive and negative ion modes. Ultraviolet (UV) absorbance was monitored using a diode array detector with a scan range of 198–800 nm, to determine which SL variants could be detected with UV.

#### Samples

To provide a range of structural variants of SLs for testing, purchased standards were used. Samples were prepared at 10 g/L in pure ethanol and filtered with a 0.4-µm polytetrafluoroethylene (PTFE) filter.

#### Analysis

##### MS1 peak picking

Data analysis was performed using MZmine from Schmid et al*.* (2023) for the MS and MS2 data and FreeStyle (ThermoFisher, USA) for the photodiode array data analysis. The molecular formula for each SL variant was selected alongside different adducts (depending on the scan mode) to search the MS1 chromatogram for likely peaks. Peaks were selected as likely candidates based on their retention time, the presence of the peak with different adducts (i.e., present in [M–H]^−^ and [M+CHO_2_]^−^ in negative mode and [M+H]^+^ and [M+NH_4_]^+^ in positive mode). A minimum intensity of 1.0 × 10^6^ counts was set for peaks to avoid false positives. In cases where the data-dependent data acquisition mode selected the adduct peaks for further collision in MS2, the MS2 spectra were collected.

##### MS2 analysis

MS2 analysis was performed in order to fragment selected suspected SL variants to produce fragmentation spectra that could be compared and analysed with in silico fragmentation tools. The MS2 spectra generated through tandem MS of the SL variants were analysed in two ways. Firstly, the data were prepared using JMP Pro 17 and spectra compared between different structural variants to find shared/unique fragment masses between structures. The relative intensity of peaks was calculated by comparing the intensity of any given peak to the highest intensity fragment. The neutral loss and/or difference from the parent ion was calculated by subtracting the m/z value from the mass of the parent ion. Spectra were compared using JMP Pro 17’s Graph Builder platform, analysing the difference in patterns between spectra with changes in structural components. This was used to identify peaks that were unique to certain structural characteristics (i.e., a peak that only appeared in all lactonic forms). Similarly, this was used to determine whether peak patterns were identical but “shifted” in mass (increasing/decreasing), providing a likely common structure between variants.

To facilitate structural characterization of the fragments, the in silico fragmentation tool MetFrag was used (Ruttkies et al*.*, 2016). MetFrag compared the experimental spectra of a given variant to in silico fragmentations of the suspected parent ion, utilizing structures provided by a custom database, and then matched the likely fragment structure based on intensity and m/z value. The fragmentation settings were configured to 5 mzppm, 0.001 ppm, and two-level candidate trees. In cases where MetFrag could not correctly identify structures, alternative forms were hypothesized using ChemSketch and the “MassSpec Scissors” tool, guided by the theoretical fragmentation schemes of de Koster et al*.* (1995) and the fragmentation patterns of other related glycoconjugates under LC-MS-ESI and tandem MS with collision induced dissociation (CID) or HCD.

##### Spectra elution order

Following confirmation of the structure, extracted ion chromatogram for the adducts of [M–H]^−^ and [M+CHO_2_]^+^ adducts was taken, selected for their high intensity in the chromatogram. The beginning and end of the peaks were determined by taking the point in time the peaks deviated (beginning) and returned (end) to the baseline, providing a time range for each structure in the chromatogram. A standard least squares model was performed in JMP 17 Pro to estimate the linear effect of form (acidic or lactonic), chain length (16–19), unsaturation (0–2), and acetylation (DA, MA C6′, MA C6″, and NA). The carbon number and unsaturation were treated as numeric continuous variables, whilst the acetylation and form were treated as categorical. To find the mean change in retention time from changing a structural component, the scaled estimates were extracted from the model for the numeric continuous factors (carbon number and unsaturation) and least square means found with a Student’s *t*-test (form) or Tukey’s test (acetylation). This allowed for the determination of predicting the change in retention time, supported by a statistically significant model.

## Results and Discussion

### Initial Identification of Sophorolipid Structural Variants via Chromatographic Spectrum

**Fig. 2.**
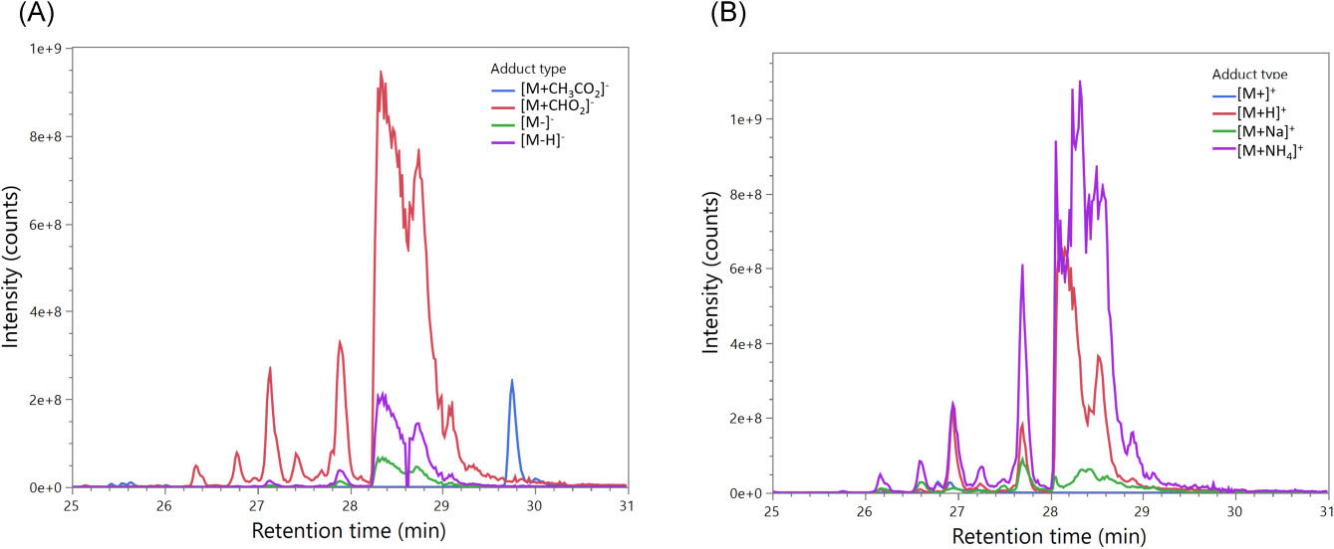
Extracted ion chromatogram of different adducts of C18:1 diacetylated lactonic sophorolipids under negative (A) or positive (B) mode mass spectrometry.

In order to separate and identify the potential SL variants present in the tested samples, reverse-phase LC (with a C18 column) was used to separate the SL variants via their relative polarity, allowing for ionization by ESI-MS to produce a mass chromatogram of time (x-axis), intensity (y-axis), and mass (z-axis). In LC-MS-ESI, the analyte undergoes ionization to form molecular ions ([M+]^+^ or [M–]^−^) formed by electron loss/gain, and adduct ions (i.e., [M+NH_4_]^+^ or [M+CHO_2_]^−^) formed by the interaction of the parent ion with atoms/molecules present in the sample solution (Kruve & Kaupmees, 2017). The resulting chromatogram is a mixture of peaks that represent single analytes that have separated during the LC process. To identify the specific peak that represents the target analyte, the mass chromatogram can be searched at the specific mass of the molecular ion/adduct ion to produce an extracted ion chromatogram. In this work, the mass of the molecular ion and different adduct ions of the target analyte/SL variant were extracted and the chromatograms compared to find peaks that shared identical retention times, pinpointing the peak that represented the target SL variant (Erngren et al*.*, 2019; Gonzalez-Riano et al., 2021). As shown in Fig. 2, this was used effectively to identify the likely peaks generated by C18:1 DA Lac. By extracting the masses for the negative ([M–]^−^, [M–H]^−^, and [M+CHO_2_]^−^, A in Fig. 2) or positive ([M+]^+^, [M+H]^+^, [M+Na]^+^, and [M+NH_4_]^+^, B in Fig. 2) modes and adducts/molecular ions, the shared peaks demonstrated that C18:1 DA Lac (and all variants) occurred between 26 and 29 min with a distinct peak shape. If a single adduct was selected to search for a variant, there is the potential for misconstruing the positioning of a target molecule (De Vijlder et al*.*, 2018). This is illustrated with the [M+CH_3_CO_2_]^+^ variants; where the other negative forms show a common peak between 26 and 29 min, [M+CH_3_CO_2_]^+^ shows a sole peak at ∼29.5 min. Similarly, in positive mode the [M+H]^+^ adduct shows three peaks at 24 min that are not the C18:1 DA lactonic form (based on fragmentation patterns established in this work). In the literature, SL variant selection is typically only presented with one adducted form with little evidence of searching for alignment with other adducts, particularly when applying the “soft” ionization of ESI where multiple adduct forms should be detected. An important consideration is that different adducts may have preference for different SL variants as adduct formation varies depending on the donor/acceptor ions in the structure, so it is important to explore all potential adducted forms of a molecule (Gonzalez-Riano et al., 2021; Mortier et al*.*, 2004).

### MS2 Spectra

In total, 37 SL variants were identified for further analysis based on the initial parent ion/adduct search of the LC-MS data, giving retention time values to search for any data-dependent acquisition tandem MS2 fragmentation that had occurred (where peaks with significant ion strength were automatically sent to MS2 for fragmentation). Thirty-six negative and 37 positive MS2 spectra were found using the [M–H]^−^ and [M+NH4]^+^ adducted parent ions (for the full list, see Supplementary Appendix 1).

Data-dependent acquisition was able to acquire tandem MS spectra for the [M–H]^−^, [M+Na]^+^, [M+H]^+^, and [M+NH_4_]^+^, the latter two showing closely matching MS2 spectra. Despite having high intensity, the [M+CHO_2_]^−^ adduct forms were not selected for top five, likely due to the high intensity increasing the noise and leading to filtering by the selection software. The focus of this work is on the [M–H]^−^ and [M+NH_4_]^+^ adducted forms. Sodiated adducts [M+Na]^+^ have been selected in some research articles as suitable adduct forms for analysis of SL fragmentation, particularly as they lead to different fragmentation patterns to protonated forms. Here, however, reliable/consistent MS2 results from this adduct could not be obtained, with no/few fragmentation matches in the in silico fragmentation tool used in this work. de Koster et al*.* (1995) similarly reported poor sodiated adducts of the lactonic SL form, so it is possible that the lactonic SL structure does not readily accept sodium adduction.

### Identified SL Structural Variants

Following the initial identification of structural variants via specific mass and confirmation with MS/MS fragmentation analysis, 37 total structural variants were identified (as summarized in Supplementary Appendix 1). Comparing to the literature, 11 variants have been characterized with LC-MS and MS/MS, including the popular C18:1 DA Lac and C18:1 DA acidic forms that are favourably produced by the wild-type *S. bombicola.* A further 11 variants have been previously identified in the literature, but do not have MS/MS applied to fully confirm the structure via fragment analysis. Finally, 15 variants identified in this work have not been reported in the literature, composed primarily of odd-chain (C17 and C19) acidic and lactonic forms. Odd-chain variants are typically rarer, as direct incorporation is lower in *S. bombicola* and fatty acids are preferentially broken down to supply even-chain fatty acids within the cell via β oxidation and *de novo* fatty acid synthesis (Asmer et al*.*, 1988). Cavalero and Cooper (2003) demonstrated some odd-chain variants with gas-chromatography mass-spectrometry (GC-MS) for C15:0, 15:1, 17:0, and 17:1 DA Lac forms; however, this required methanolysis of the SL structures and extraction of the lone hydroxy fatty acid chains. This work provides the first extensive analysis of the fragmentation behaviour of odd-chain SL structures via LC-MS/MS.

### In Silico Fragmentation and Fragmentation Pathways

#### Process for determining the fragmentation characteristics of sophorolipid variants

To elucidate the structure of a given molecule, the mass value provided by MS1 is insufficient and fragmentation by tandem MS is required to provide fragments that represent the core structural components that confirm the identity of the molecule. The typical approach in metabolomics/lipidomics is to take the experimental fragmentation spectra and compare against a database containing the suspected identity of the target molecule, using automated analysis tools to suggest likely structures and fragment structures. Popular databases including the Human Metabolome Database (HMDB), MassBank, and Metlin hold thousands of potential structures and spectra with associated MS and MS2 data for comparison; however, no spectra are publicly available for any SL variant. In the literature, LC-MS or MS2 analysis of SLs typically does not provide the full spectra data, limiting the comparability of different MS applications. As such, scientists exploring SL MS analysis must find another process to target and identify fragments in their experimental spectra that proves the structural composition of the SL in question. The goal of this work was to take MS2 spectra generated for different structural variants and identify their structural composition and understand the process of how different variants fragment.

As limited data are available, the MS2 spectra of tentatively identified structural variants were analysed using in silico computational fragmentation with MetFrag. Through this tool, the experimental MS2 spectra were compared against topological fragments generated from suggested structures (Blaženović et al*.*, 2018; Cauët et al*.*, 2021; Heller et al*.*, 2015; Krettler & Thallinger, 2021; Ruttkies et al*.*, 2016).

To provide these structures, a custom database of SL variants was created with InChi descriptors (see the “Data Availability” section), as there are a limited number of SL structures available in popular structural databases such as PubChem and ChemSpider, with a general focus on the most “popular” forms (i.e., C18:1 fatty acid-containing forms). As MetFrag is not based on mechanistic understanding of fragmentation, it is possible to create ions that do not follow a “logical” process of fragmentation. To provide a “common sense” approach to support and filter the findings of MetFrag, the fragmentation process of SLs (suggested by other researchers) and similar structures (glycoconjugates) under CID and HCD were considered, underpinned with fragmentation theory associated with ESI MS2 methods (De Vijlder et al*.*, 2018; Grabarics et al*.*, 2022; Tsugawa et al*.*, 2016). CID occurs under a “slow heating” method where the ion in question goes under a high number of collisions to slowly accumulate enough energy to fragment. These fragmentations usually occur through charge retention or charge migration fragmentations as the molecule undergoes redistribution of its charge (Demarque et al*.*, 2016; Gross, 1992). Whilst not as common, HCD can undergo the same fragmentation pathways as CID, but is also capable of reaching higher energies, leading to direct bond cleavage and the production of smaller fragments through sequential fragmentation, which can be used to improve identification (Sartori et al*.*, 2014).

The seminal work of de Koster et al*.* (1995) established the first fragmentation schemes for acidic and lactonic SLs using experimental data from CID fragmentation—exploring the likely fragmentation process of the sugar moieties and lipid chains in protonated, deprotonated, and sodiated forms. This work has been heavily referenced in later works and has acted as a guide for the majority of MS-based SL investigations in the literature (including in this work); however, new fragments/fragment pathways have not been suggested beyond this original work. This is an interesting finding given that fragmentation can differ greatly between ionization mode, analyser, activation methods, collision energy, and adduct forms (Dardouri et al*.*, 2021; Nuñez et al*.*, 2001; Ribeiro et al*.*, 2012). With the advent of in silico fragmentation tools and modern MS processes/equipment, the SL fragmentation process needs updating to provide improved fragmentation data for SL variants that aids in confident identification with LC-MS.

With the limited amount of elucidated SL fragmentation pathways, the fragmentation of related structures was explored to provide more of an underpinning of likely fragmentation, looking at groups that contain sugar moieties and/or lipid chains. Glycoconjugates (carbohydrates covalently linked to other functional groups such as proteins and lipids) are well-researched and characterized molecules through MS fragmentation, due to their relevance in biological systems and can provide an insight into how sugar moieties may fragment in MS. For example, the most common fragmentation of glycans in glycopeptides and glycolipids occurs at either the glycosidic bonds (referred to as B, C, X, and Y types in the literature) or across the ring structure (A and X types), with the product ions containing either the reducing portion (A, B, and C ions) or the nonreducing portion/aglycon (X, Y, and Z ions, see Supplementary Appendix 7 for more details). CID and HCD most often cause glycosidic cleavages to yield B and Y ions in glycopeptides (depending on the collision energy), whilst X-link cleavages can be improved through metal cation coordination or use of negative ionization (Cao et al*.*, 2014; O'Brien & Brodbelt, 2013; Wuhrer et al*.*, 2007; Zeng et al*.*, 2022). This sugar loss is usually associated with retro-heteroene reactions such as McLafferty rearrangements or through dehydration via remote hydrogen rearrangement and subsequent retro-Diels–Alder fragmentation (Demarque et al*.*, 2016; Grabarics et al*.*, 2022; Niessen, 2011; Tsugawa et al*.*, 2016). Examples of B, Y, and X fragmentation are found in oligosaccharides (Zhang & Julian, 2014), gangliosides (O'Brien & Brodbelt, 2013), and glycolipids (Grabarics et al*.*, 2022; Murphy & Axelsen,^ 2011^), providing a likely fragmentation to occur in SLs (see Supplementary Appendix 7). The fragmentation of glycans will produce characteristic mono- or disaccharide fragments called oxonium ions that are used to identify the composition of the glycan chain, for example by producing hexose oxonium ions (Hex+) at a mass of 163.0633 [Hex+H]^+^ and 145.057 [Hex–H_2_O]^+^ Da, which would similarly be expected to be produced in the NA or MA forms of the SLs (Pirro et al*.*, 2021; Toghi Eshghi et al*.*, 2016). Conversely, there is little understanding on the fragmentation of glycans with acetyl groups such as those on C6′ or C6″ of the SL structure. Whilst some acetylated oxonium ions have been produced in fragmentations of glycopeptides, these are usually N-linked acetyl groups, such as N-acetyl hexosamines, which present different bond dissociation energies and fragmentation under CID and HCD, making comparison difficult (Domon & Costello, 1988).

Lipid analysis with MS is similarly popular due to its importance in biology and can provide information on the expected fragmentation of the fatty acid chain of the SL. Whilst SLs are considered glycolipids, they are more accurately labelled as fatty acyl glycosides, which are under-researched when compared to other glycolipids such as glycoglycerolipids, glycosphingolipids, and glycosylphosphatidylinositols (Bailey et al*.*, 2021; Crittenden et al*.*, 2017; Fahy et al*.*, 2009; Grabarics et al*.*, 2022). In these groups, and other fatty acid-containing components, the lipids typically fragment under charge remote fragmentations, leading to shortening of the long chain up to the allylic carbon (adjacent carbon to the double bond), which aids in identification of the double bond position (Gross, 1992; Harvey, 2005; Hsu & Turk, 1999; Wang et al., 2013).

#### Characteristic differences of MS2 spectra when altering structural form of sophorolipids

Based on the initial MetFrag identification, verified by comparing against current knowledge in SL and glycoconjugate fragmentation, a series of fragmentation pathways have been suggested for lactonic and acidic SLs containing variation in the unsaturation, fatty acid chain length, and acetylation form. The simplified pathways are shown in Figs. 5, 6, 7, and 8; however, examples of acetyl-specific and unsaturation-specific groups are available in the “Data Availability” section. From this, common fragmentation patterns and major identifiers for changes in the SL structure can be identified. As an overview, there are four main areas where the variant fragmentation change is either “simple” or “complex.” In simple changes, alteration of the fatty acid chain length and fatty acid unsaturation cause minor alterations in the m/z value of fragments that contain structures of the fatty acid region—generally causing a “shift” to the left or right in m/z. More complex changes appear in the alteration of ring structure (from acidic to lactonic) or acetylation, where the whole fragmentation pattern is altered and shared fragment masses appear.

**Fig. 3.**
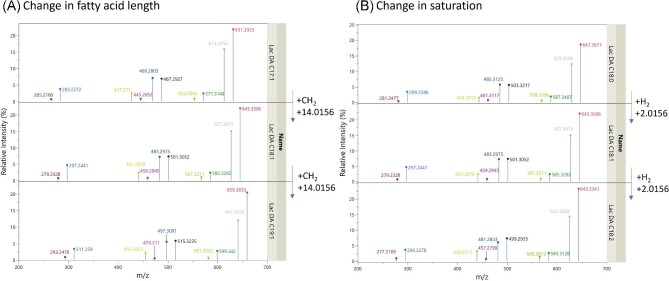
An example of the “simple” changes that can occur with alteration of sophorolipid structure in tandem mass spectrometry (MS2) spectra, with a change in chain length (A) and unsaturation (B). MS2 generated via deprotonated [M–H]^−^ adducts of lactonic sophorolipids. Related structures are presented in matching colours.

Simple changes can be found when comparing a change in the fatty acid chain length, where similar fragments (containing the whole/part of the fatty acid chain) see the fragment pattern increase/decrease with reflected by minor alterations in the m/z value of fragments that contain structures of the fatty acid region—seeing the fragment patterns shifting left or right. This is shown in Fig. 3, where fragments of a related structure (i.e., the hydroxy fatty acid chain fragment) change by 14.0156 Da when increasing/decreasing fatty acid chain length (reflecting the addition/subtraction of CH_2_) or a change of 2.0156 Da as the unsaturation is changed (addition/subtraction of H_2_). This indicates that, regardless of chain length/saturation, the fragmentation process is not greatly altered, starting at an identical position of the fatty acid, either at the “start” by the carboxylic acid or “end” by the hydroxylation position and the subsequent fragments will simply be one carbon longer or shorter as chain length changes.

**Fig. 4.**
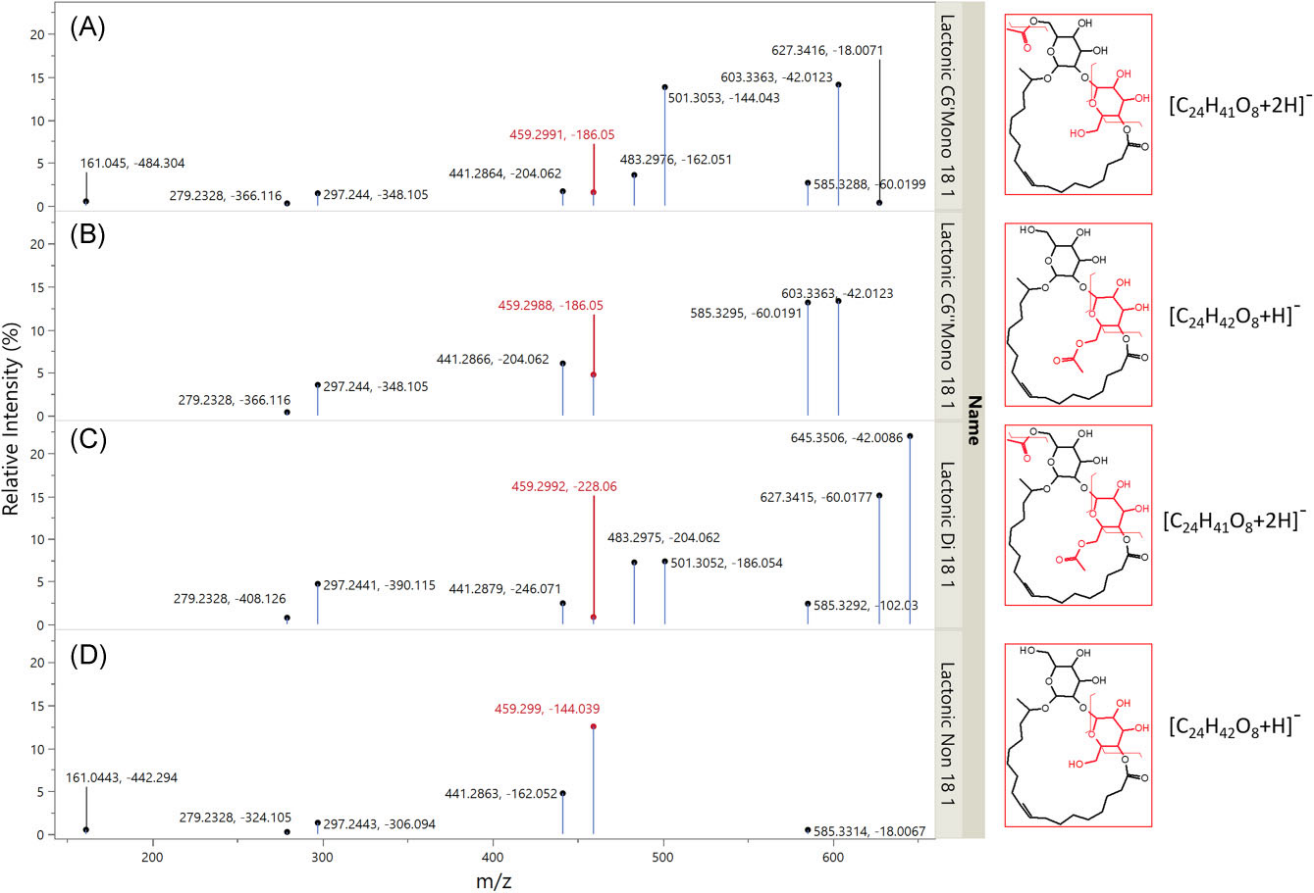
An example of the “complex” changes that can occur with the alteration of the acetylation of the C18:1 lactonic sophorolipids. Tandem mass spectrometry spectra generated via [M+NH_4_]^+^ adducts of C18:1 lactonic sophorolipids varying in acetylation. Value labels = m/z value, m/z loss from parent ion. Full structures and neutral loss components (marked red) are shown for the 459.299-Da mass fragment for each acetylated form.

More complex changes in the mass spectra appear when attempting to compare the change in ring form (acidic or lactonic), acetylation form (NA, MA, or DA), and acetylation position (C6′ or C6″ monoacetylation). Unlike fatty acid chain length and unsaturation, alteration of these parts of the SL structure leads to the appearance of shared mass fragments—which could quickly lead to the conclusion that (a) the fragments generated are identical structures or (b) the selected molecules are not unique in their structure. This is shown in Fig. 4, where the negative MS2 spectra of different acetylated forms of lactonic C18:1 SL show a series of shared mass fragments. If the neutral loss (mass difference from the parent ion) and the potential structural ID are considered, it becomes clear that this mass corresponds to different final structures; the fatty acid and C6′ sugar structure shared between the NA and C6″ MA variant (black structures in a and c in Fig. 4) and the fatty acid chain and DA C6′ sugar between the DA and C6′ MA form (black structures of b and d in Fig. 4). Additionally, the fragment that has been lost (neutral loss, marked red) to achieve these structures is different between each acetylation form—as shown in the structures in Fig. 4. The DA form (c) has lost a fragment of 228.0 Da, whilst the C6′ MA form (a) has lost 186.05 Da. The addition or subtraction of hydrogen, associated with the aforementioned charge retention/migration fragmentation, further complicates the identification process as structures that normally present different neutral masses are protonated/deprotonated to equivalent ion mass. In addition, the intensities of these shared mass fragments are different, as structural variants can exhibit different ionization efficiencies, leading to variations in their signal intensities in the mass spectrum (Liigand et al*.*, 2021).

#### Identified fragmentation pathways of sophorolipid structures

Following the identification of prospective peaks for the SL variants, the MS2 spectra of the [M–H]^−^ and [M+NH4]^+^ adducts were analysed and the fragmentation patterns were established using the in silico fragmentation tool MetFrag and available literature on SL fragmentation, particularly from the work of de Koster et al*.* (1995), who first described prospective fragmentation pathways in SL. Suggested pathways for fragmentation have been generated, and in cases where there are two possible identities for a given fragment, two theorized pathways have been suggested. Some fragments contain protonation, either sourced from the solvent, adduct, or intramolecular proton transfer; however, this work has avoided trying to predict where the source of hydrogen is from. The hydrogen atom's journey can involve several steps and intermediate species, making it difficult to trace its exact path, especially if multiple pathways are possible. The isotopic equivalence of hydrogen in any position means that, based solely on MS data, it is impossible to differentiate between hydrogen atoms originating from different positions in a molecule. Whilst techniques such as density functional theory can be used, it is outside the scope of this work. In addition, the distinction of peaks with identical mass as ω-1 or ω terminal forms was not possible; all peaks produced near identical MS2 spectra (in terms of unique mass fragments) between them. Distinction of the hydroxylation group is usually identified by techniques such as H-NMR and GC-MS (Cavalero & Cooper, 2003; Davila et al*.*, 1993; de Koster et al*.*, 1995; Nuñez et al*.*, 2001).

A key consideration, which is repeated through the suggested fragmentation pathways, is identifying the position of the acetyl group, in particular the MA sugars, as the acetylation can occur on C6′ or C6″. Identification of these forms is difficult as they both have identical mass, so must be fragmented in such a way that the position of the acetyl group can be established through unique fragments/fragment patterns. Unique fragments between the MA forms can be hard to predict; both forms are capable of producing a nonacetylated and acetylated sugar and, without the attachment of other functional groups associated with the start or end of the fatty acid tail in the fragment structure, determining whether the fragment is from a C6′ or C6″ position is difficult. This limitation similarly carries itself over to the in silico fragmentation tool used here; when the MS2 spectra of suspected MA forms are inputted to MetFrag, the tool often has difficulty distinguishing the “most likely”/logical fragment, instead providing all possible fragments that could form from the structure. As with all in silico fragmentation tools, supplementing the identifications with knowledge of the expected fragmentation pathways is essential to form sensible fragmentation pathways. The fragmentation pathways of acidic and lactonic forms under negative and positive ionization are described to provide an understanding of the expected fragmentation process when applying MS2, identifying fragments and fragment groups that can be used to give confidence in the structural identity of a potential variant. Full pathways for each individual form found in this work are available in the “Data Availability” section.

##### Acidic sophorolipids—negative ionization

**Fig. 5.**
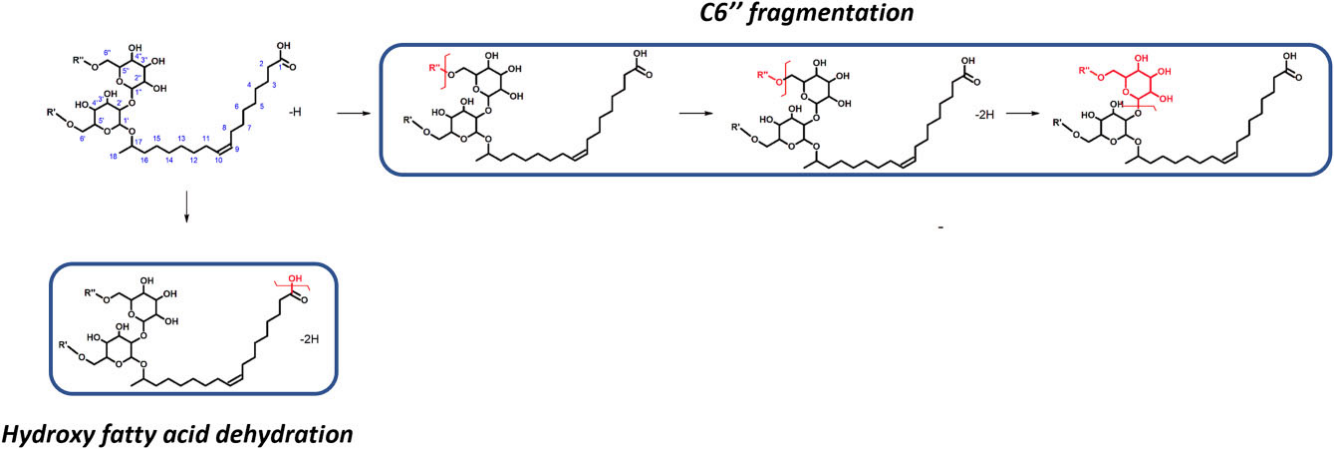
Suggested fragmentation pathway for acidic sophorolipids under negative ionization with HCD from protonated [M–H]^−^ adducts. The fatty acid of the structure is presented as C18:1 as an example, with the functional groups on C6′ and C6″ shown as R′ or R″ to represent that the groups can be acetylated or nonacetylated during fragmentation.

Negative ionization is not a typical choice for analysing SL fragmentation, as saccharides are more readily ionizable to accept protons instead of losing them. However, under negative ionization the acidic SLs fragmented heavily, forming a large number of fragments in the 0- to 300-Da range, demonstrating a breakdown of the parent ion into smaller components. These smaller fragments were harder to discern/identify using MetFrag and/or referencing the expected structures from the literature, which produced a relatively simple fragmentation pathway of only five fragments, fewer than those found in the other forms in this work. As shown in Fig. 5, two pathways were formed, with the simple dehydration of the carboxylic acid end of the fatty acid tail and the sequential fragmentation of the C6″ sugar.

Under negative mode, we suggest that sugar loss first occurs on the C6″ sugar of the acidic SLs, via cleavage of the C6′/C6″ glycosidic, leading to the loss of C6″ sugar from the SL structure (Fig. 5, C6″ fragmentation), which is in line with the expected loss of the reducing sugars from glycoconjugates (Rožman, 2016). The neutral mass loss can be used to identify the acetylation of the C6″ sugar, producing a neutral loss of an NA sugar fragment of 163.0603 Da, shared between the C6′ and NA form and an acetylated sugar fragment of 205.0172 Da, shared between the C6″ and DA form. The subsequent products are a fatty acid chain with an acetylated or nonacetylated C6′, which can be used to confirm the acetylation form of the C6′ sugar (by accounting for the mass of the fatty acid fragment). Only one fragmentation was seen to occur on the fatty acid chain, with dehydration of the hydroxyl group from the carboxylic end for a loss of 18.0106 Da, which could be used to determine the fatty acid carbon length and unsaturation. Whilst dehydration could occur on any of the available hydroxyl groups across the SL structure fatty acids, the loss here matches the expected single dehydration of the carboxyl groups seen in fatty acids under negative ionization (Murphy & Axelsen,^ 2011^). If the dehydration were to occur from the sugar-bound hydroxyls, then sequential dehydration from the other sugar-bound hydroxyls would be expected, producing a pattern of sequential neutral losses of 18.0105 Da, which was not found (Wan et al*.*, 2013).

##### Acidic sophorolipids—positive ionization

**Fig. 6.**
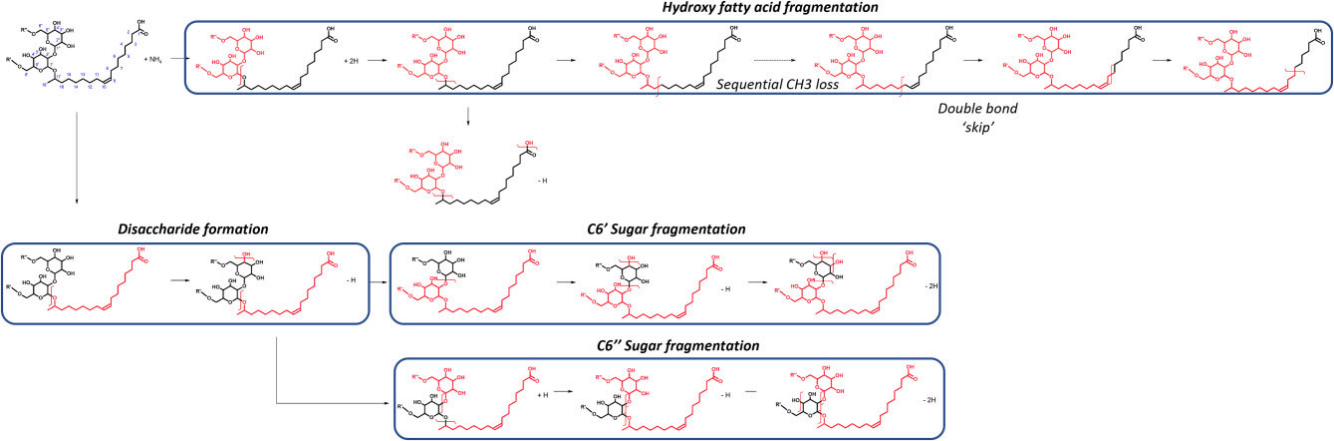
Suggested fragmentation pathway for acidic sophorolipids under positive ionization with HCD from [M+NH4]^+^ adducts. The fatty acid of the structure is presented as C18:1 as an example, with the functional groups on C6′ and C6″ shown as R′ or R″ to represent that the groups can be acetylated or nonacetylated during fragmentation.

Typically, the ionization process only results in one of the fragment “halves” becoming ionized and subsequently detected (as a B or Y ion in glycoconjugates, see Supplementary Appendix 7). Interestingly, the positive ionization of acidic SLs led to the production of both B and Y ions. This observation was also captured in de Koster et al*.* (1995), where both fragments could gain protonation and eventual detection under LC-MS. This produced a more detailed fragmentation pathway—as both fragments could be detected in the MS2 spectra. The suggested fragmentation pathway for acidic SLs under positive ionization with HCD from [M+NH_4_]^+^ adducts is shown in Fig. 6. The main pathways were hydroxy fatty acid fragmentation and disaccharide formation, with two theoretical C6′ or C6″ sugar fragmentation.

Unlike negative ionization, where fragmentation occurred at the C6″/C6′ glycosidic bond, the fatty acid/C6′ sugar glycosidic bond was cleaved, creating a sophorose fragment unique to acidic SL forms under positive ionization. This was similarly found in Nuñez et al*.* (2001) and Ribeiro et al*.* (2012) via atmospheric pressure chemical ionization; however, this was the primary peak in their spectra, presumably due to the different form of ionization used. This sophorose unit made distinction of the DA and NA groups simple, with a product ion of 409.1344 or 325.1123 Da, respectively. However, the C6′ and C6″ MA forms produced identical mass fragments (367.1129 Da) as the mass did not change depending on where the acetyl group was positioned.

Whilst spectra peaks were found that suggested the sophorose was further fragmented at the remaining glycosidic bond, it is unclear whether the fragments were from a C6″ sugar (with no retained oxygen from the glycosidic bond) and the C6′ sugar (which retained the oxygen from the C6′/C6″ glycosidic bond), so a theorized pathway was suggested for either a C6′ or C6″ (Fig. 6). For example, the fragmentation of the MA sophorose produces a fragment of 205.0698 Da, which could be attributed to an acetylated C6′ or acetylated C6″, supported by its presence in the spectra of both the C6′ and C6″ MA acidic SLs. Similarly, the NA forms produce peaks at 163.0591 Da, which could be sourced from either sugar. Whilst the C6″ sugar does contain an additional hydroxyl group (theoretically producing a fragment 18.0105 Da larger than the C6′), the retention of the oxygen from the glycosidic bond, alongside an additional proton during positive ionization, makes the mass identical. For glycoconjugates, the most common fragmentation involves retention of the glycosidic oxygen atom to the nonreducing sugar and fragmentation of the reducing sugar via neutral loss, with a preference for B and Y ions, meaning it is far more likely to form a sugar fragment without the glycosidic oxygen attached (Hoffmann et al*.*, 2018; Rožman, 2016). However, glycopeptides have shown the ability to produce two possible fragments of the glycan containing or not containing the glycosidic O bond that can be produced, similar to this work (Yu et al*.*, 2016). Numerous research articles state that the peak should be assigned to the C6″ sugar (without the glycosidic bond); however, we found the peaks appeared in both the C6′ and C6″ MA forms (de Koster et al*.*, 1995; Gupta & Prabhune, 2012; Nuñez et al*.*, 2001). Sequential dehydration of the C6′/C6″ fragments is shown, which is common in the elimination of the oxonium ions with HCD (Wuhrer et al*.*, 2007). It was not possible to distinguish the acetylation position from the positive ionization mode alone, and identification via negative ionization was required. This highlights the utility of choosing both ionization forms and the potential challenges in identifying fragments of bioproducts where repeating units produce hard to discern fragment structures. In theory, lower collision energy may provide fragmentation of the C6′/C6″ glycosidic bond instead, making identification of the acetylation position easier.

The final fragmentation pathway identified was produced from the hydroxylated fatty acid fragment following sophorose loss; the hydroxylated fatty acid (with glycosidic oxygen) undergoes oxygen loss from the glycosidic bond, producing a fatty acid that sequentially loses CH_2_ (14.028 Da) (Fig. 6). The full hydroxy/non-hydroxy fatty acid can be used to discern the carbon length/saturation of the SL fatty acid component. The position of the carbon–carbon double bond can be found by a “skip” in the sequential CH_2_ loss, as the underlying charge remote fragmentation is unable to overcome the energy requirements to fragment the allylic (opposing carbons) to the double bond (de Koster et al*.*, 1995; Gross, 2000; Hsu & Turk, 2008; Jensen et al., 1985). Using this, the position of the double bonds at C9 and C12 was confirmed for the mono- and diunsaturated acidic SLs, which is the expected double bond position for SLs produced via vegetable oils such as rapeseed oil. One limitation of this approach was that the intensity was low in the MS2 spectra, so some peaks of the post-double bond carbons were not found in every SL. This is presumably due to the low relative concentration of some of these compounds in the samples tested, which should be taken into account when performing targeted analysis with MS. Alternatively, higher collision energies could promote the fragmentation of the carbon bonds in the fatty acid structure, leading to higher intensities.

##### Lactonic sophorolipids—negative ionization

**Fig. 7.**
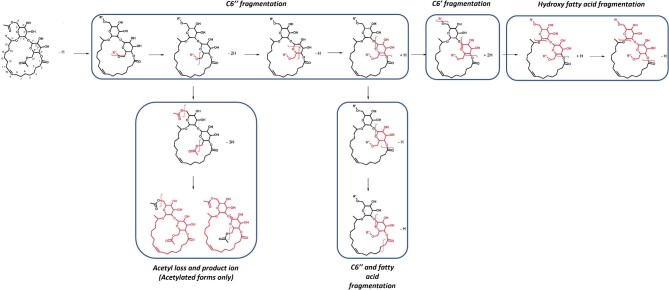
Suggested fragmentation pathway for lactonic sophorolipids under negative ionization with HCD from [M–H]^−^ adducts. The fatty acid of the structure is presented as C18:1 as an example, with the functional groups on C6′ and C6″ shown as R′ or R″ to represent that the groups can be acetylated or nonacetylated during fragmentation.

The fragmentation pathways for lactonic SLs under negative ionization (with an [M–H]^−^ adduct) are shown in Fig. 7. Firstly, the acetylated functional groups are fragmented (primarily from the C6″ sugars), indicated by a neutral loss of 42.090 Da and the detection of a fragment at 42.090 Da. This provided information on the presence of acetylated sugars, as this peak did not appear in the NA forms (Fig. 7, acetyl loss and product ion). This neutral loss was similarly reported in de Koster et al*.* (1995) in deprotonated adducts; unfortunately there are not many other works that report lactonic SL fragmentation under negative ionization, so information is limited.

As with the positive ionization, the position of the acetylation on the MA forms could be determined by comparing the spectra with the DA or NAs. A peak cluster was found that was 42.090 Da higher in the DA and MA C6′ forms when compared to the NA and MA C6″, which represented the production of a hydroxylated fatty acid with a C6′ sugar attached. Using C18:1 forms as an example, the C6′ produced a fragment (501.305 Da) identical to those in the DA spectra—showing a hydroxylated fatty acid with an acetylated C6′ sugar attached. Neutral loss from this fragment then matched with the NA (144.040 Da) demonstrating the loss of an NA C6″ sugar—confirming the position of the acetyl group on the C6′. Inversely, the C6″ shared a product fragment with the NA (459.299 Da), representing a fatty acid with an NA C6′ sugar, with a neutral loss of 186.050 Da, with the DA showing the loss of an acetylated C6″ sugar. Unlike positive ionization, there was clearer support that the loss occurred on the C6″ as the subsequent loss of the carboxyl group and fatty acid chain at C1 (Fig. 7, C6″ and fatty acid fragmentation) could only occur if the C6″ had been lost first. Despite the lack of negative ionization studies of SLs in the literature, this fragmentation is very useful for confirming the position of sugar loss.

Following the loss of the C6″ sugar, the C6′ is then released to form a hydroxylated fatty acid with a mass value that can be used to confirm the length and unsaturation of the SLs chain. Unlike positive ionization, it was not possible to see the sequential shortening of the chain from losses of the carbons, which limited the abilities of negative ionization for determining the position of any unsaturated carbons. Exploration of different collision energies in the negative ionization method may be needed to determine whether this process is possible with lactonic SLs, as it has been reported in the literature for unsaturated fatty acids (Tomer et al., 1983).

##### Lactonic sophorolipids—positive ionization

**Fig. 8.**
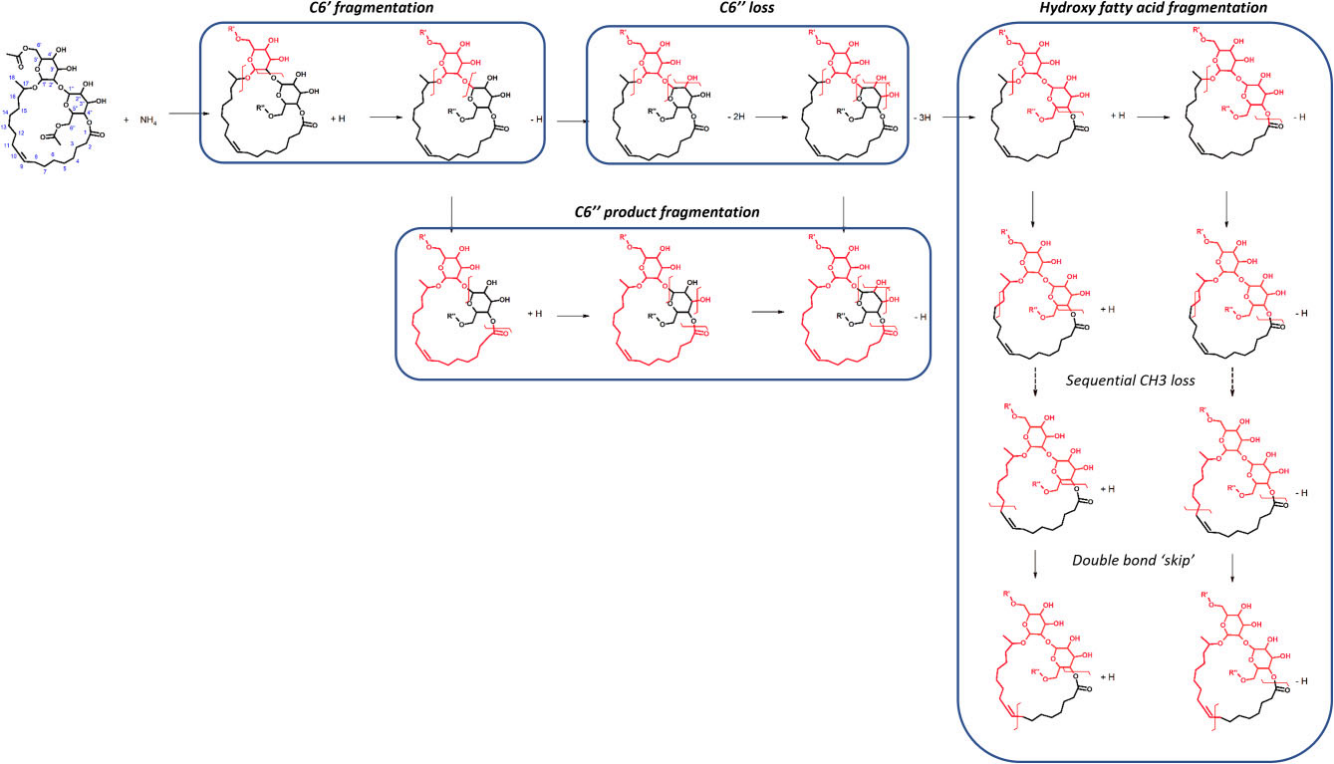
Suggested fragmentation pathway for acidic sophorolipids under positive ionization with HCD from [M+NH4]^+^ adducts. The C6″ fragmentation only occurs on the acetylated forms. The fatty acid of the structure is presented as C18:1 as an example, with the functional groups on C6′ and C6″ shown as R′ or R″ to represent that the groups can be acetylated or nonacetylated during fragmentation.

Determining the position of the acetylated sugars for the MA forms was a different challenge to those found in the acidic SLs. Whilst the acidic SLs would be expected to fragment from the reducing/external sugar first, the binding of the fatty acids to the C6′ and C6″ sugars closes this structure off, making it unclear as to which sugar should fragment first. This confusion was further exacerbated when spectra of lactonic SLs were tested with MetFrag, which was unable to distinguish which sugar form is the most likely to fragment and determine the structure of fragments in the MS2 spectra. Whilst the literature provided a good guide for the expected fragmentations of glycoconjugates with “free” reducing sugars, there was limited information on forms that may have a cyclical structure such as those found in lactonic SLs and no information on how they are expected to fragment under MS. With the guidance of the work of de Koster et al*.* (1995), we determined that the fragmentation occurred on the C6′ sugar first (Fig. 8, C6′ fragmentation), with subsequent hydrolysis of the hydroxyl groups of the C6″ sugar fragment (Fig. 8, C6″ loss).

The indication that this fragmentation occurred on the C6′ sugar first was shown in the mass spectra of the MA forms. The two MA peaks demonstrated two different spectra, primarily in a repeating cluster of fragments that showed a sequential loss of 18.015 Da. The first (retained) peak started this cluster 42.0106 Da mass higher than the second (i.e., for the C18:1 group the masses were 485.3120 and 443.3014 Da). This sequential hydroxyl (−OH) loss gave strong indication that these peaks involved the C6′ or C6″ sugars. When compared alongside the DA and NA forms, identical fragment patterns (and relative intensities) were paired between the DA and the first peak in the MA and the NA and the second peak of the MA. de Koster et al*.* (1995) similarly found two fragments at 485.3120 and 443.3014 Da in C6′ and C6″ MA C18:1 Lac forms and identified that these fragments were formed by loss of the C6′ group and that the difference of 42.0106 Da was associated with the presence/lack of the acetyl functional group. This becomes clear when comparing the product ion/neutral losses of the peaks of the MA forms with the DA and NA. Using C18:1 forms as an example, the first MA peak shared a fragment of 467.298 Da with the DA form, indicating that the product ion was a hydroxylated fatty acid with a single acetylated sugar. The identity of the last fragment was confirmed by shared neutral loss with the NA (197.091 Da)—indicating that the lost fragment was an NA C6′ sugar. The combination of the product and neutral loss masses confirmed that the first peak was the C6″ MA Lac form. Inversely, the same rules could be applied to the second peak; the product peak (664.390 Da) matched those of the NA, demonstrating a hydroxylated fatty acid with a single NA sugar whilst the neutral loss (239.102 Da) matched the DA peak demonstrating the loss of an acetylated C6′ sugar, confirming the form of a C6′ MA Lac form. We know from these results that the loss of the sugar can only occur on the C6′ or the C6″ first—if it were possible for either to interchangeably fragment first then both MA forms would show identical fragments (of the nonacetylated or acetylated sugars) and distinction would be impossible. The original proposal from de Koster et al*.* (1995) that fragmentation occurs on the C6′ has been used to structure the fragmentation pathways proposed here, but it should be noted that this is the only literature article that suggests a fragmentation pathway for the lactonic SLs under positive ionization—more work is needed to confirm that the likely fragmentation position would be from the C6′ sugar first, either through applying advanced in silico fragmentation tools, although attempts with CFM-ID and MetFrag in this work were unsuccessful (not reported). Mass spectrometry alone is not unambiguous enough of a tool to identify the exact structure of a given fragment and the simplicity of the SL structure means there can be potentially hundreds of potential structures that can be applied to a given fragment mass, even if there is a high confidence in the fragment formula.

Following the loss of the C6′ sugar group, the remaining C6″ sugar undergoes hydrolysis of the 2″, 4″, and 5″ hydroxyl groups, with an additional peak in the C6′ MA and NA forms representing the loss of the 6″ hydroxyl group—again helping to distinguish between the C6′ and C6″ MA forms (Fig. 8, C6″ product fragmentation). In the MA C6″ and DA forms, there was a cluster of fragments (with the same relative intensities) that was indicative of the release of the C6″ sugar as a separate fragment, with subsequent loss of two hydroxyl groups (at 205.0704, 187.0606, and 169.0493 Da). Finally, the remaining hydroxylated fatty acid chain underwent the sequential loss of carbons until allylic carbons, identical to that seen in the acidic forms. Two separate pathways were found representing the sequential loss of carbons from a hydroxylated fatty acid with the oxygen bond from the glycosidic bond and one without (Fig. 8, hydroxy fatty acid fragmentation).

#### Effect of sophorolipid structural components on retention time

Establishing how SL variants typically separate gives a better understanding for unknown structures and provides prospective identification based on the retention of variants. It also aids in their isolation with methods such as prep HPLC, where they must be isolated/purified for further analysis (i.e., nuclear magnetic resonance) and physical property testing (i.e., critical micelle concentration measurements). The key goal of this work was to better understand how structural changes affect the elution of SL variants when run through a commonly used HPLC analytical method (RP, C18 column with a gradient elution of acetonitrile/water). In particular, a focus was placed on trying to identify where acidic and lactonic forms may “cross over” during their elution as other structural components change (acetylation, chain length, and unsaturation), which can lead to misidentification of the variant structure when using less specific detection methods that are more commonly found/accessible (UV, ELSD, and charged aerosol detector (CAD)). This focus was brought due to the tendency of the literature to describe the separation of lactonic and acidic SLs as a clearcut “acidic first, lactonic second” mode of separation, which seemed unlikely given the other areas where the structures can alter (i.e., fatty acid chain length, unsaturation, and acetylation) and affect the separation.

**Fig. 9.**
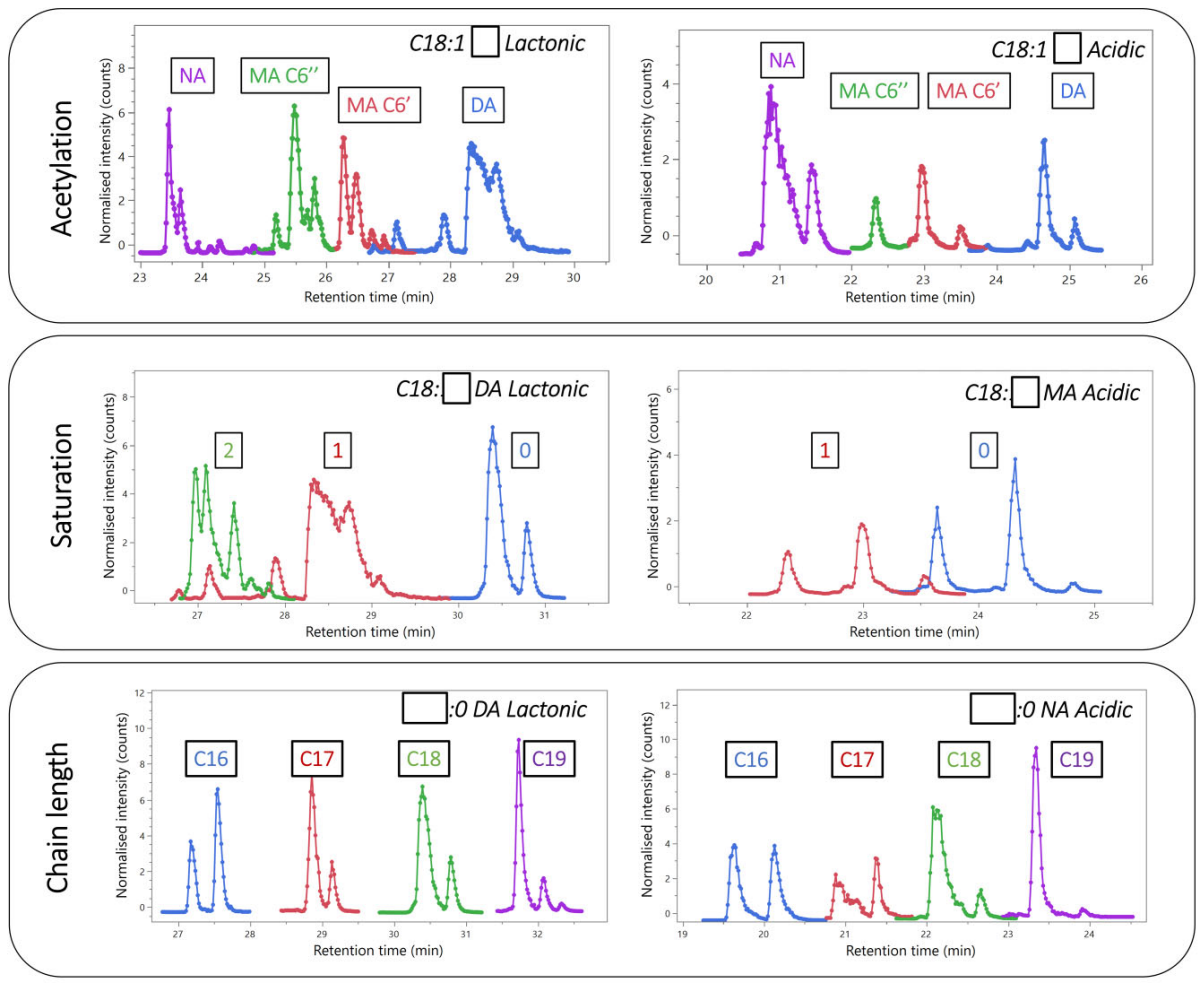
The effect of altering the structural components of a lactonic (left) or acidic (right) sophorolipid on separation in reverse-phase HPLC (C18 column).

For a reverse-phase HPLC gradient elution method such as that used in this study, the separation is primarily dependent on the hydrophobicity of the compound—as more hydrophobic compounds interact with the stationary phase to a great extent and more hydrophilic compounds elute earlier from the column. As the proportion of acetonitrile in the mobile phase increases, the strength of the hydrophobic interactions with the stationary phase decreases and the compounds are eluted and detected. It is possible to predict the hydrophobicity of a compound to provide an estimate of possible elution order (such as those predicted from the structures by ChemSketch in this work), but other effects such as steric hindrance, structure size/shape, and specific interactions (dipole–dipole, dipole–π) can also alter the retention time of a specific compound, making the prediction difficult (Neue et al., 2006; Tanaka et al*.*, 1993; Wu et al., 2008). The focus of this work was to establish the separation characteristics of 37 SL variants under the same test conditions, to provide a guideline for the expected elution order of a wide array of SLs for future works. Comparison to the current literature where C18 RP HPLC is used for SL detection is possible; however, these typically focus on a small subset of the potential structures, most typically around the C18 acidic and lactonic forms. Additionally, variations in column size, length, mobile phase chemistry, and gradient—all affect the final retention time of the analytes, making comparisons between the works difficult to establish an expected elution order of a wide array of variants (such as the range of chain length, unsaturation, and acetylation found here). A summary of the effect of altering the structure of acidic and lactonic SLs is shown in Fig. 9, highlighting how the change of acetylation, unsaturation, and chain length causes different retention times in the chromatogram. The chromatogram was generated by negative mode ionization and the peaks found at the masses of the [M–H]^−^ adducts. The same patterns were found in the positive ionization forms. As shown, the retention time of both lactonic and acidic SLs increases with increasing acetylation, chain length, and unsaturation.

As shown in Fig. 9, multiple peaks of identical m/z value were found to have eluted closely when searching the expected masses of given SL variants (in MS1), with two major peaks closely eluting. The two large peaks were believed to be the ω and ω-1 terminal forms of the variants; however, analysis of the MS2 spectra for these peaks demonstrated closely matching peak patterns, which made it impossible to discern any unique fragments that could pinpoint the hydroxylation position. Further identification of the position has been confirmed with methods including nuclear magentic resonance (NMR) and gas chromatography of acid-hydrolysed SLs; however, this was outside the scope of the work (Davila et al., 1994; Nuñez et al*.*, 2001; Van Bogaert et al*.*, 2011). It has been widely reported that the ω-1 terminal form elutes first in C18 RP-HPLC and is the most commonly produced form, so the assumption was made that the large eluting peak was that of the ω-1 terminal form, followed by the ω terminal form (Ashby et al., 2008; Davila et al., 1994; Jiménez-Peñalver et al*.*, 2020; Nuñez et al*.*, 2001; Van Bogaert et al*.*, 2011). Additional peaks were found to appear before and after these peaks, being particularly prominent in variants with high intensity/concentration in the sample, which we theorize may be rarer variants in the SL structure associated with changes in the position of the esterification in lactonic forms from the C4′ to the C6′ or C6″ position (Ciesielska et al*.*, 2016).

Acetyl groups are hydrophobic in nature, meaning the addition onto the C6′ and/or C6″ sugars (replacing the hydrophilic hydroxyl groups) of the SL structure will subsequently lead to greater interaction with the stationary phase of the HPLC column and increased retention with the stationary phase, which is similarly shown in other molecules such as oligopeptides (Evjenth et al*.*, 2009; Sedláčková et al*.*, 2023). Interestingly, the addition of an acetyl group did not produce an equal increase in retention time, rather this is dependent on where it was added (see Supplementary Appendix 4). Between the different forms, retention time increased an average of 1.06 ± 0.33 min (NA to MA C6″), 0.96 ± 0.32 min (MA C6″ to MA C6′), and 2.07 ± 0.297 min (MA C6′ to DA). The greatest shift in retention was associated with acetylation to the C6′ sugar, either from an NA to MA C6′ (2.017 ± 0.33 min) or MA C6″ to DA (3.03 ± 0.30 min, respectively), indicating that acetylation of that position greatly contributes to the hydrophobicity and retention behaviour in SLs; however, this may be linked to a number of factors including steric hindrance, shape/size change, or new interactions with the stationary phase that are not possible with the C6″ MA form. In general, the elution order of NA > MA > DA is found in other literature applying C18 HPLC to SLs (Dardouri et al*.*, 2021; Jiménez-Peñalver et al*.*, 2020; Ribeiro et al*.*, 2012). The only work in disagreement is that of Davila et al. (1994), where they found the elution order to dependent on the chain structure of the SL, with acidic forms eluting MA > NA > DA and lactonics as the expected NA > MA > DA, despite using similar column chemistry, mobile phase, and gradient to this and other works. Specific focus on the elution of the C6′ and C6″ elution is rarer in the literature; however, the order of C6″>C6′ MA has been shown in long- (>C22) and medium-chain (C16–C18) forms (Dardouri et al*.*, 2021; Ribeiro et al*.*, 2012). Predicted LogP values are significantly higher for C6′ variants in the acidic forms, but do not display a statistically significant difference for lactonics (see Supplementary Appendix 2), suggesting an interaction between the form and acetylation for hydrophobicity; however, this trend was not carried over when comparing the retention times of the peaks; changing from MA C6″ to MA C6′ always increases retention time, regardless of it being acidic or lactonic (see Supplementary Appendix 3).

The structure of the fatty acid tail was found to have the same effect, regardless of it being in a lactonic or acidic form. In all cases, decreased unsaturation and increased chain length led to an increase in the retention time, as the hydrophobicity of the SL structure increased. Studies of SLs (typically in the C16 to C18 and non- to diunsaturated range) all demonstrate the effect on retention time shown in the study (Ashby et al., 2006, 2008; Nuñez et al*.*, 2004, 2001; Ribeiro et al*.*, 2012). Cases of targeted synthesis of long-chain (>C20) and triunsaturated (C18:3) lactonic and acidic forms show that the retention time is increased or reduced respectively (Ribeiro et al*.*, 2015, 2012). Identical behaviours are seen in the glycolipid biosurfactant rhamnolipids, as shorter fatty acids and higher unsaturation lead to decreased hydrophobicity and earlier elution (Behrens et al*.*, 2016). Similarly, free and methylated fatty acids display the same behaviour on C18 HPLC (Carvalho et al*.*, 2012; Lin et al., 1995; Mansour, 2005). The change in unsaturation and chain length both showed a linear relationship with the retention time of the SL variant, with a decrease in unsaturation leading to a peak shift of 1.51 ± 0.91 min (see Supplementary Appendix 5) and an increase in chain length (per 1C addition) having a 1.97 ± 0.17-min increase. Whilst this linearity may extend to further changes in chain length, extreme cases of considerably short- (<C10) and long- chain (>C30) variants may require consideration of alteration to the gradient conditions and run time to achieve suitable resolution (Abhyankar et al*.*, 2021; Xu et al*.*, 2019; Zerhusen et al*.*, 2020).

Throughout the literature there is a common statement that acidic SLs always elute before their lactonic counterparts when using a C18 RP-HPLC gradient method, which often leads to chromatograms with “acidic” and “lactonic” sections that are distinctly separated (Ashby et al., 2008; Jiménez-Peñalver et al*.*, 2020). Whilst it is true that acidic forms do generally separate earlier due to their lower hydrophobicity, the chain form is not the only structural component that contributes to the retention time, meaning it is only logical that there must be a form of acidic SLs that possess a greater retention than some forms of lactonic. Given that the shift from acidic to lactonic increases the retention time in this work by an average of 3.41 ± 0.25 min (see Supplementary Appendix 5), the combination of the chain length, unsaturation, and acetylation that positively increase retention time should be able to overcome that difference in retention time, leading to an acidic variant appearing later than a lactonic. As shown in Supplementary Appendix 8, there is a region of acidic and lactonic variants that “cross over”; the first appearance of a lactonic peak (based on measures of intensity at the specific m/z value to the variant) occurs before all of the acidic forms have eluted, with the C18:1 DA variant eluting last for the acidic. Lactonic forms with low chain length, no acetylation, or monoacetylation on C6″ or high unsaturation appear in the same region as acidic forms with longer chains (C18 or C19), lower unsaturation (1), and deacetylation and C6′ monoacetylation, supporting the hypothesis that an acidic variant can be eluted later than a lactonic via the combination of other structural components that contribute positively to retention. The findings here emphasize the need to avoid the assumption that the retention of acidic and lactonic variants can be separated into distinct groups. Practically, the majority of SL analyses will be/is performed on nonspecific (UV, ELSD, and CAD) detection methods, often with standards/samples rich in a wide array of potential SL variants. Relying on the assumption that lactonic and acidic SLs distinctly separate in chromatograms can introduce the risk of misinterpreting results and inaccurately assessing sample compositions. This misconception holds significant repercussions in process development, where the distinct physiochemical differences between lactonic and acidic SLs may necessitate tailored production processes. Ensuring the precision of SL identification is pivotal in establishing an effective feedback loop for process refinement. While MS is a valuable tool for discerning SL variants based on mass, its accessibility is limited by factors such as cost and complexity. For those utilizing the nonspecific methods mentioned, it is important to be cautious with peak assignments and be mindful of the crossover issues described here.

#### Public database and interactive dashboard

The majority of published data is not supplemented with LC-MS data and data that are published are often static images, which may not include the specific number of resolution/decimal places needed to distinguish the variant type. Whilst commercial software allows for the integration of popular MS libraries (i.e., MassBank, KEGGS, LipidMaps, PubChem, and HMDB), SL forms are not found. Structural forms of SL variants can be found in LipidMaps (six structures) and PubChem (four structures at the time of writing), but structural information is not as valuable compared to access to physical mass spectra data. As SL research advances and new/rare structural forms become increasingly common, access to spectral data for comparison and structure identification is essential in order to accelerate the understanding and innovation in a compound that has huge potential to disrupt the chemical surfactant market. In comparison with the accessibility of spectral data of compounds such as lipids, fatty acids, protein, and peptides, SLs (and biosurfactants) are significantly behind (Kind et al*.*, 2018). As such, the MS1 chromatogram (of adducted forms) and MS2 spectra from this study have been made publicly available via JMP Public, with the hopes that the data can be used to aid in the research of SLs with LC-MS. A dashboard for the MS2 spectra allows for selection of the structural components and provides an annotated map of the spectra for each structural variant found in this work, with fragment structures annotated on those identified. The structures found in this work have also been uploaded to PubChem for access via in silico fragmentation tools such as MetFrag, and the SMILES and ChemDraw structures are available for download.

The application of MS can vary in the settings applied to fragment the compound of focus, particularly in the collision energy, collision form, mass analyser type, and adduct form, which can lead to different fragmentation forms than those found in those work. These differences can help to further elucidate the structures of SL variants and potentially identify key fragments that are essential for identification. The distribution of this data is essential to grow the available information for SL identification and should be highly encouraged.

## Conclusion

The large degree of possible natural variations in the structure of SLs inherently complicates the identification process when applying techniques to separate and identify these molecules, such as HPLC-MS, as sufficient separation and specific/unique fragmentation is required to gain certainty on the identification. This certainty is key in all stages of SL production, informing process optimization, product applications, and regulatory filings to name a few. The presence of identical masses in structural isomers (MA forms) and mislabelling of adducted forms can quickly lead to misidentification and reduce confidence in the quality and accuracy of analytical work. The work here provides a novel and comprehensive analysis of 37 SL variants, compared under identical conditions, to highlight the potential pitfalls and key areas of consideration that must be made when engaging in analytical identification of SLs with LC-MS. Of the 37 variants found, 15 have not been reported in the literature, 11 partially reported (found via mass with no MS/MS analysis), and 11 reported with MS/MS, providing new reference spectra and identities of novel SL variants.

This work provides data and insight to enable better understanding of how SLs separate in the most commonly used method (RP-HPLC with a C18 column and acetonitrile–water gradient elution) and confidently identify a wide array of variants by comparing the unique and shared fragments that are found between them in MS2 analysis. Using the identified variants, the positive effect of lactonization, acetylation, unsaturation, and chain length on the retention time of variants and dispelling the common misconception that acidic and lactonic forms have clearly distinct “groups” when eluting. Positive and negative fragmentation pathways were identified, along with unique pathways (particularly for distinguishing MA isomers). The culmination of this work is the release of the chromatogram and MS2 data in a publicly available dashboard, which will help other researchers quickly and confidently identify a wide array of SL structural variants. The hope is that this work will be developed on and supplemented with more experimental data with different variations in the MS form, settings, and adducts in order to further improve the quality of research in SL identification.

## Supplementary Material

kuae035_Supplemental_File

## Data Availability

The database of sophorolipid chromatograms and MS2 fragmentations is freely available at JMP Public (see Supplementary Information).

## Notes

Datasets, including the SMILES dataset used in MetFrag, are freely available at https://pure.manchester.ac.uk/admin/workspace.xhtml?openEditorId=4e1fbbbc-3d0f-4b76-96b0-4026fdf4a170&family=dataset.

## Notes

All structures and fragmentation pathways are available as images in the attached data files in the Supplementary section.

## Notes

All structures and fragmentation pathways as ChemSketch files for each sophorolipid variant found in this work are also available at https://pure.manchester.ac.uk/admin/workspace.xhtml?openEditorId=bb4cf88a-7504-4ece-8489-529a6bff930c&family=dataset.

## Notes

Structures have also been uploaded to PubChem at https://www.ncbi.nlm.nih.gov/pcsubstance?term=%22UOM%20Winterburn%20Research%20Group%22%5BSourceName%5D%20AND%20hasnohold%5Bfilt%5D.
