# Supplementary material for: Uncovering the fragmentation and separation characteristics of sophorolipid biosurfactants with LC-MS-ESI
Source: J Ind Microbiol Biotechnol. 2024 Sep 26;51:kuae035. doi: 10.1093/jimb/kuae035 (PMC11484030; doi:10.1093/jimb/kuae035)
Supplement: kuae035_Supplemental_File [file kuae035_supplemental_file.docx]

**Uncovering the fragmentation and separation characteristics of sophorolipid biosurfactants with LC-MS-ESI**

Benjamin Ingham^1^, Katherine Hollywood^2^, Phavit Wongsirichot^1^, Alistair Veitch^3^, James Winterburn^1^*

^1^ Department of Chemical Engineering, The University of Manchester, Oxford Road, Manchester, M13 9PL, United Kingdom

^2^ Manchester Institute of Biotechnology, Department of Chemistry, University of Manchester,

Manchester, M1 7DN, United Kingdom

^3^ Holiferm Ltd., Unit 15, Severnside Trading Estate, Textilose Road, Manchester, M17 1WA

* Corresponding author

Email: james.winterburn@manchester.ac.uk, Phone: +44 161 529 3013

**Supplementary Information**

MS2 Spectra Dashboard –

<https://public.jmp.com/packages/V5MChV0113hH0X3dm3KFX>

MS1 Chromatogram Dashboard –

<https://public.jmp.com/packages/ZCzwzJP066b94ZQSjb5PD>

| **Reported?** | **Chain Structure** | **Acetylation** | **Acetylation Position** | **Chain length** | **Saturation** | **References** |
| --- | --- | --- | --- | --- | --- | --- |
| Not reported | Acid | Di |  | 17 | 0 |  |
| Not reported | Acid | Non |  | 16 | 1 |  |
| Not reported | Acid | Non |  | 17 | 0 |  |
| Not reported | Acid | Non |  | 17 | 1 |  |
| Not reported | Acid | Non |  | 19 | 0 |  |
| Not reported | Acid | Non |  | 19 | 1 |  |
| Not reported | Lactonic | Di |  | 19 | 0 |  |
| Not reported | Lactonic | Di |  | 19 | 1 |  |
| Not reported | Lactonic | Di |  | 19 | 2 |  |
| Not reported | Lactonic | Mono | C6' | 16 | 0 |  |
| Not reported | Lactonic | Mono | C6' | 16 | 1 |  |
| Not reported | Lactonic | Mono | C6'' | 16 | 0 |  |
| Not reported | Lactonic | Mono | C6'' | 16 | 1 |  |
| Not reported | Lactonic | Mono | C6'' | 17 | 0 |  |
| Not reported | Lactonic | Non |  | 18 | 0 |  |
| Reported, fully characterised | Acid | Di |  | 18 | 1 | (Ribeiro *et al.*, 2012; Hu and Ju, 2001; Kurtzman *et al.*, 2010; Ratsep and Shah, 2009; Ma *et al.*, 2011; Jiménez-Peñalver *et al.*, 2019; Dardouri *et al.*, 2021; Adu *et al.*, 2023; De Clercq *et al.*, 2021) |
| Reported, fully characterised | Acid | Mono | C6' | 18 | 1 | (Ribeiro *et al.*, 2012; de Koster *et al.*, 1995; Kurtzman *et al.*, 2010; Ma *et al.*, 2011; Dardouri *et al.*, 2021; Adu *et al.*, 2023; De Clercq *et al.*, 2021) |
| Reported, fully characterised | Acid | Mono | C6'' | 18 | 1 | (Ribeiro *et al.*, 2012; de Koster *et al.*, 1995; Kurtzman *et al.*, 2010; Ma *et al.*, 2011; Dardouri *et al.*, 2021; Adu *et al.*, 2023; De Clercq *et al.*, 2021) |
| Reported, fully characterised | Acid | Non |  | 18 | 1 | (Ribeiro *et al.*, 2012; de Koster *et al.*, 1995; Kurtzman *et al.*, 2010; Ma *et al.*, 2011; Dardouri *et al.*, 2021; Adu *et al.*, 2023; De Clercq *et al.*, 2021) |
| Reported, fully characterised | Lactonic | Di |  | 16 | 0 | (Ashby, Solaiman and Foglia, 2008; Ribeiro *et al.*, 2012; Nuñez *et al.*, 2001; Hu and Ju, 2001; Jiménez-Peñalver *et al.*, 2019; Dardouri *et al.*, 2021) |
| Reported, fully characterised | Lactonic | Di |  | 18 | 0 | (Ashby, Solaiman and Foglia, 2008; Ratsep and Shah, 2009; Jiménez-Peñalver *et al.*, 2019; Dardouri *et al.*, 2021) |
| Reported, fully characterised | Lactonic | Di |  | 18 | 1 | (Ashby, Solaiman and Foglia, 2008; Ribeiro *et al.*, 2012; de Koster *et al.*, 1995; Kurtzman *et al.*, 2010; Jiménez-Peñalver *et al.*, 2019; Dardouri *et al.*, 2021) |
| Reported, fully characterised | Lactonic | Di |  | 18 | 2 | (Ashby, Solaiman and Foglia, 2008; Ribeiro *et al.*, 2012; Ratsep and Shah, 2009; Ma *et al.*, 2011; Jiménez-Peñalver *et al.*, 2019; Dardouri *et al.*, 2021) |
| Reported, fully characterised | Lactonic | Mono | C6' | 18 | 1 | (Ribeiro *et al.*, 2012; de Koster *et al.*, 1995; Kurtzman *et al.*, 2010; Ma *et al.*, 2011; Jiménez-Peñalver *et al.*, 2019; Dardouri *et al.*, 2021) |
| Reported, fully characterised | Lactonic | Mono | C6'' | 18 | 1 | (Ribeiro *et al.*, 2012; de Koster *et al.*, 1995; Kurtzman *et al.*, 2010; Ma *et al.*, 2011; Dardouri *et al.*, 2021) |
| Reported, fully characterised | Lactonic | Non |  | 18 | 1 | (de Koster *et al.*, 1995; Kurtzman *et al.*, 2010) |
| Reported, no MS/MS performed | Acid | Di |  | 18 | 0 | (Ribeiro *et al.*, 2012; Adu *et al.*, 2023) |
| Reported, no MS/MS performed | Acid | Mono | C6' | 18 | 0 | (Ma *et al.*, 2011; Adu *et al.*, 2023; De Clercq *et al.*, 2021) |
| Reported, no MS/MS performed | Acid | Mono | C6'' | 18 | 0 | (Ma *et al.*, 2011; Adu *et al.*, 2023; De Clercq *et al.*, 2021) |
| Reported, no MS/MS performed | Acid | Non |  | 16 | 0 | (Adu *et al.*, 2023) |
| Reported, no MS/MS performed | Lactonic | Mono | C6' | 18 | 0 | (De Clercq *et al.*, 2021) |
| Reported, no MS/MS performed | Lactonic | Mono | C6'' | 18 | 0 | (De Clercq *et al.*, 2021) |
| Reported, no MS/MS performed | Lactonic | Di |  | 16 | 1 | (Ashby *et al.*, 2013) |
| Reported, no MS/MS performed | Lactonic | Mono | C6' | 17 | 0 | (Ashby *et al.*, 2013) |
| Reported, no MS/MS performed | Acid | Non |  | 18 | 0 | (Ma *et al.*, 2011; Adu *et al.*, 2023; De Clercq *et al.*, 2021; Kurtzman *et al.*, 2010) |
| Reported, no MS/MS performed | Lactonic | Di |  | 17 | 0 | (Cavalero and Cooper, 2003; Ashby, Solaiman and Foglia, 2008; Nuñez *et al.*, 2001) |
| Reported, only analysed with methanolysis and GC-MS | Lactonic | Di |  | 17 | 1 | (Cavalero and Cooper, 2003) |

Appendix 1: Identified sophorolipid variants found in this study


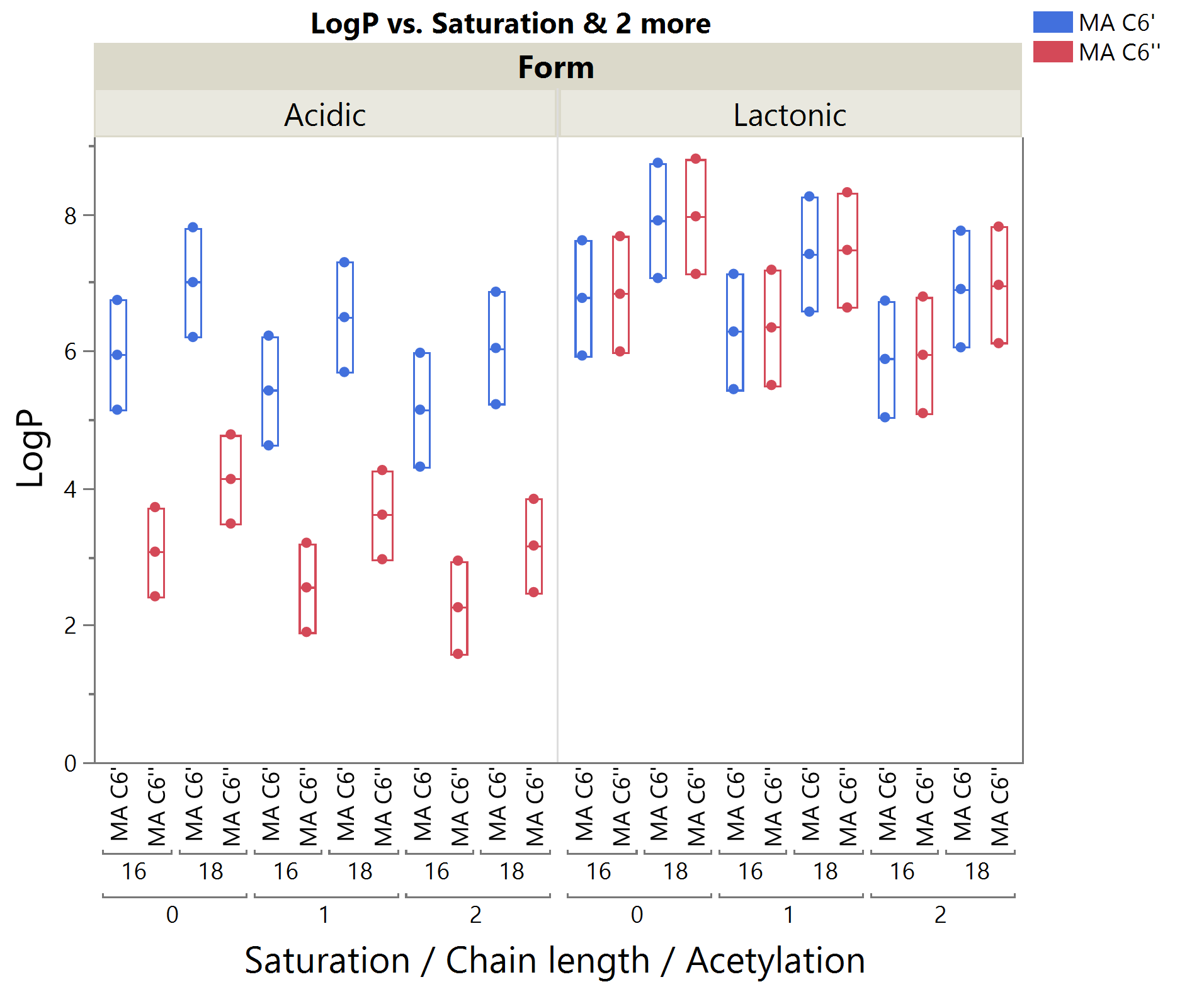


Appendix 2: Comparison of theoretical LogP values with sophorolipids with different structural variants


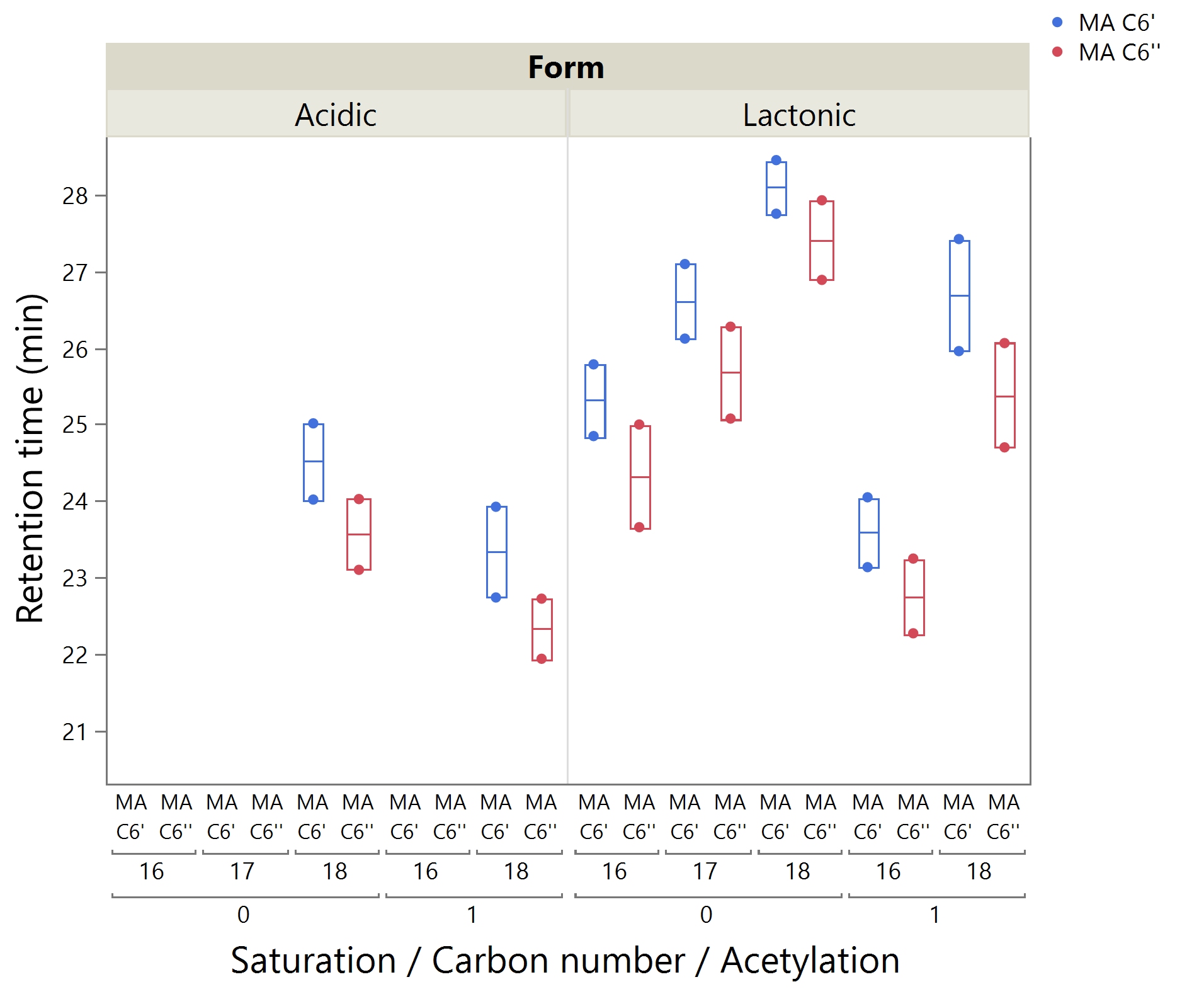


Appendix 3:Comparison of the retention times of identified sophorolipid structural variants.

| **Level** | **- Level** | **Difference** | **Std Err Dif** | **Lower CL** | **Upper CL** | **p-Value** |
| --- | --- | --- | --- | --- | --- | --- |
| Di | Non | 4.089883 | 0.3007525 | 3.296211 | 4.883555 | <.0001* |
| Di | MA C6'' | 3.031217 | 0.2968391 | 2.247873 | 3.814561 | <.0001* |
| Di | MA C6' | 2.072155 | 0.2968391 | 1.288811 | 2.855499 | <.0001* |
| MA C6' | Non | 2.017728 | 0.3334700 | 1.137717 | 2.897740 | <.0001* |
| MA C6'' | Non | 1.058666 | 0.3334700 | 0.178655 | 1.938678 | 0.0121* |
| MA C6' | MA C6'' | 0.959062 | 0.3299598 | 0.088314 | 1.829810 | 0.0253* |

| **Level** | **- Level** | **Difference** | **Std Err Dif** | **Lower CL** | **Upper CL** | **p-Value** |
| --- | --- | --- | --- | --- | --- | --- |
| Lactonic | Acidic | 3.425467 | 0.2434493 | 2.93872 | 3.911961 | <.0001* |

Appendix 4: Least square means Tukeys HSD test comparing the retention time from altering the acetylation and chain structure of the sophorolipid structures. a = 0.050

| **Term** | **Scaled Estimate** | **Std Error** | **t Ratio** | **Prob>\|t\|** |
| --- | --- | --- | --- | --- |
| Intercept | 24.66191 | 0.109683 | 224.85 | <.0001* |
| Form[Acidic] | -1.712733 | 0.121725 | -14.07 | <.0001* |
| Form[Lactonic] | 1.7127333 | 0.121725 | 14.07 | <.0001* |
| Acetylation[Di] | 2.2983138 | 0.171165 | 13.43 | <.0001* |
| Acetylation[MA C6'] | 0.2261588 | 0.199186 | 1.14 | 0.2605 |
| Acetylation[MA C6''] | -0.732903 | 0.199186 | -3.68 | 0.0005* |
| Acetylation[Non] | -1.791569 | 0.202094 | -8.87 | <.0001* |
| Carbon number | 1.9695141 | 0.167416 | 11.76 | <.0001* |
| Saturation | -1.506271 | 0.191242 | -7.88 | <.0001* |

Appendix 6: Scaled Estimates from the linear regression model of sophorolipid structure affect on retention time


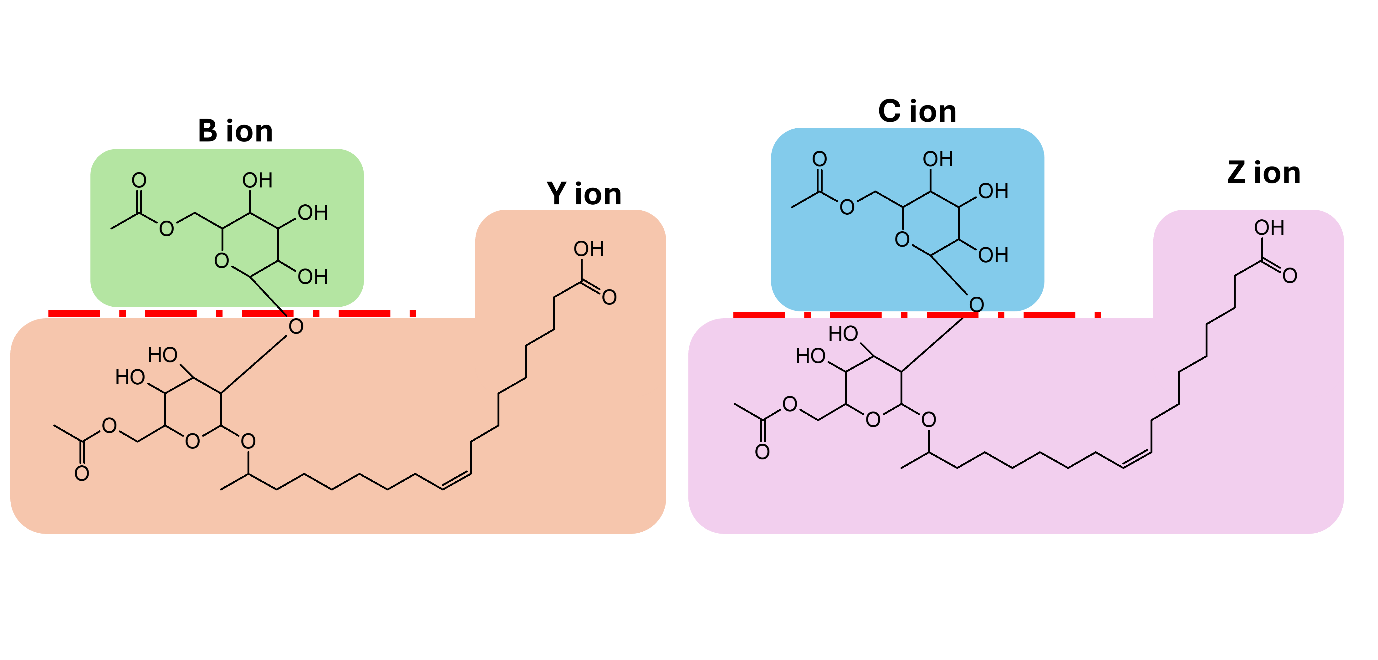


Appendix 7: Demonstration of the possible ion types that can be formed by the fragmentation of the C6'' sugar with Acidic C18:1 diacetylated sophorolipid form as an example. B and C ions represent the terminal (non-reducing) sugar with (C ion) or without (B ion) the glycosidic oxygen. Z and C ions are the complementary counterparts containing the reducing sugars with (Y ion) or without (Z ion) the glycosidic oxygen.


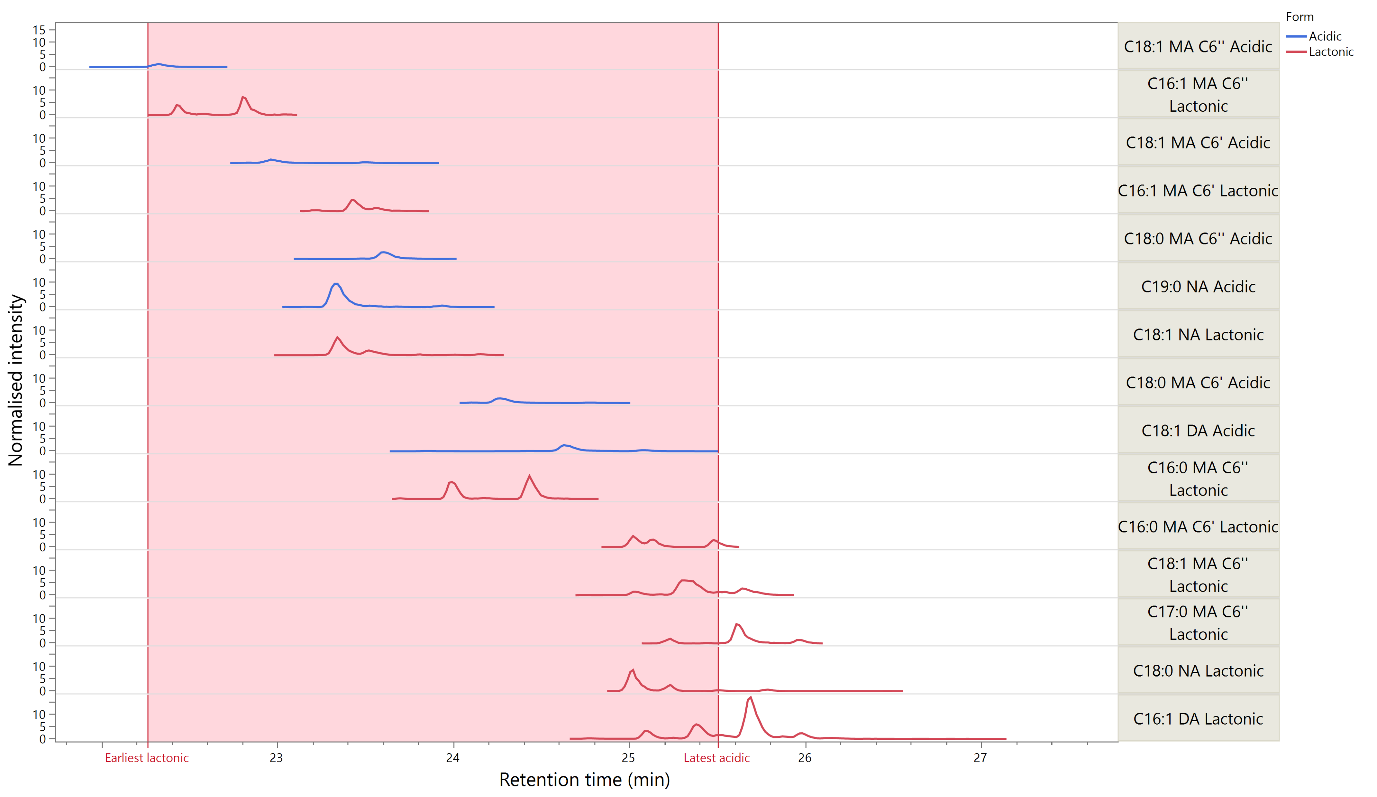


Appendix 8: The extracted chromatograms of lactonic and acidic structural variants with closely eluting peaks. Peaks are separated in order of earliest detected signal.
